# Estimating Student Attrition in School-Based Prevention Studies: Guidance from State Longitudinal Data in Maryland

**DOI:** 10.1007/s11121-023-01533-1

**Published:** 2023-05-17

**Authors:** Angela K. Henneberger, Bess A. Rose, Yi Feng, Tessa Johnson, Brennan Register, Laura M. Stapleton, Tracy Sweet, Michael E. Woolley

**Affiliations:** 1 University of Maryland School of Social Work, Baltimore, USA; 2 University of Maryland College Park, College Park, USA

**Keywords:** Attrition, Study design, School-based prevention, Program evaluation, Longitudinal data

## Abstract

Attrition is a critical concern for evaluating the rigor of prevention studies, and the current study provides rates of attrition for subgroups of students and schools who are often sampled for prevention science. This is the first study to provide practical guidance for expected rates of attrition using population-level statewide data; findings indicated that researchers using K-12 school-based samples should plan for attrition rates as high as 27% during middle school and 54% during elementary school. However, researchers should consider the grade levels initially sampled, the length of follow-up, and the specific student characteristics and schools available for sampling. Postsecondary attrition ranged from 45% for bachelor’s degree seekers to 73% for associate degree seekers. This practical guidance can help researchers to proactively plan for attrition in the study design phase, limiting bias and increasing the validity of prevention studies.

**Supplementary Information:**

The online version contains supplementary material available at 10.1007/s11121-023-01533-1.

Prevention scientists are often interested in understanding the effects of a specific program or policy on student outcomes, and school-based evaluation studies often implement randomized controlled trial (RCT) designs to limit selection bias and estimate causal effects. When RCT designs are not practical, observational designs focused on following students longitudinally over time and quasi-experimental designs that aim to equate students in two or more comparison conditions are implemented. Attrition in RCT and observational designs is defined as nonresponse or unobserved outcome data resulting from skipping or missing items and/or failure to participate in data collections. Data missingness due to attrition can be a serious threat to study validity and potentially limits the power to detect significant effects (Deke et al., 2017; Foster & Fang, 2004; Greenberg & Barnow, 2014; What Works Clearinghouse, 2020). The goal of the current study was to extend the results of Rickles et al. (2018) to provide practical guidance on expected rates of attrition to help prevention scientists plan school-based studies.

The prevention and education fields are increasingly calling for follow-up studies to examine the long-run impacts of programs and policies (see Watts et al., 2019), as evidenced by recent additions to the Institute of Education Sciences (IES) request for applications for efficacy studies focused on long-term follow-up. However, attrition becomes particularly problematic when studies follow students over multiple years, and a recent systematic review of 28 multi-year school-based evaluation studies with student-, teacher-, or school-level random assignment that included student-level outcomes reported a median overall attrition rate of 24% (interquartile range = 14 to 34%; Rickles et al., 2018). Few of these studies followed students for more than three academic years, and on average, such studies were found to lose approximately 15% of the baseline sample to attrition each year (interquartile range = 10 to 20%). Demack and colleagues (2021) reported a mean student-level attrition rate of 19% in their broad analysis of 82 RCTs and cluster RCTs conducted in the UK.

Attrition is critical for determining the rigor of school-based evaluations. The What Works Clearinghouse (WWC), an initiative of the US Department of Education’s IES, evaluates research studies that examine the effectiveness of educational programs, policies, practices, and interventions. A study with high attrition can meet the WWC’s rating of *Meets WWC Group Design Standards with Reservations* if the study can show that after attrition, the remaining intervention and comparison group members were similar on baseline characteristics*.* A study with low attrition is expected to have low bias and can meet the WWC’s highest possible rating of *Meets WWC Group Design Standards without Reservations.*

In the UK, the Educational Endowment Foundation (EEF) determines the rigor of school-based evaluations, determining data and inference quality for impact evaluations using a measure that considers whether adequate data are collected from all groups in a manner that is sufficient to make valid and reliable inferences (Maxwell et al., 2021). Demack and colleagues (2021) conducted a broad analysis of 82 RCTs and cluster RCTs conducted in the UK published prior to January of 2019. They reported that 79 of the 82 impact evaluations included information on attrition in their evaluations.

Attrition is primarily a problem when it leads to bias in the effect estimates of educational outcomes (i.e., problems with internal validity). However, few studies have focused on quantifying the degree of bias that is associated with attrition because estimation of attrition bias, by definition, depends on data from students who are no longer participating in the research study. Weidmann and Miratrix (2021) leveraged administrative data from the EEF in England linked to a census of publicly funded schools and students, offering the unique opportunity to examine the post-randomization outcomes for students who would have normally been lost to follow-up. They reported that the typical estimate of attrition bias from 10 RCTs was 0.015 effect size (ES) units, with no estimate greater than 0.034 ES. While small, they determined that the bias was nonignorable and found evidence against the *missing at random* assumption (see Little & Rubin, 2019).

Additionally, attrition is problematic because it can reduce the study’s observed sample size below the targeted sample size used in the design phase. Statistical power analyses are used to determine the target sample size for a particular minimum detectable effect size (MDES), but attrition, if not properly estimated and accounted for in MDES calculations, can result in an underpowered study. Planning for long-term follow-up and incorporating attrition estimates into MDES calculations at the start of evaluation studies require adequate empirical benchmarks for plausible expected attrition rates. Recent research aimed at providing such benchmarks estimated attrition by examining student mobility rates in four nationally representative longitudinal surveys conducted by the National Center for Education Statistics (NCES). Rickles and colleagues (2018) reported percentages of students leaving their initial school ranging from 12% over the kindergarten to first grade transition to 46% for the first grade through fifth grade period and varying by critical student- and school-level characteristics.

Here, we extend the study conducted by Rickles and colleagues (2018) to estimate rates of student attrition using statewide population-level administrative data from the state of Maryland, which is a small mid-Atlantic state with demographics that are similar to the nation as a whole. We track student movement over time between schools, districts, and out-of-the-state public school system. Using population-level data allowed us to examine attrition rates across the full grade range during K-12 and the full period of postsecondary to make inferences comparing school, district, and state attrition rates. Additionally, using statewide data, we were uniquely able to observe outcomes for students who attrited from their school or district but stayed within the state public school system. The first goal of the current study was to provide empirical benchmarks for expected attrition rates at the student population level, including attrition rates for elementary school, middle school, high school, and postsecondary students, and for specific student subgroups and school types that are often of interest for prevention scientists, such as special education students, English language learners, urban schools, and students in schools with high concentrations of students who are eligible for free/reduced price meals. The second goal of the current study was to conduct outcome analyses to understand the association between attrition and student outcomes, including academic performance, high school graduation, college enrollment, and workforce outcomes.

## Method

### The Maryland Longitudinal Data System (MLDS)

The Maryland Longitudinal Data System (MLDS) is Maryland’s repository for longitudinal administrative data linked at the student level. This study used linked records from three state agencies. The Maryland State Department of Education (MSDE) provided K-12 student data for all students for all enrollments throughout the entire academic year and summer for all students attending public schools in Maryland. The Maryland Higher Education Commission (MHEC) provided college student data for postsecondary institutions of higher education. The Maryland Department of Labor provided workforce data for students who become employed at a Maryland employer who is subject to filing quarterly Unemployment Insurance (UI) records. The federal government (including the military), certain non-profit and religious organizations, and private contractors (self-employed individuals) do not participate in quarterly UI filing. Data for out-of-state college enrollments and degrees are obtained by MSDE through the National Student Clearinghouse (NSC; Dynarski et al., 2015). NSC data are provided for students who attended 12th grade at a public high school in Maryland.

The MLDS Center links data across state agencies, years, and data files within state agencies using a state-assigned student ID (SASID), which is a unique student identifier used by MSDE, the social security number, date of birth, first name, last name, and a demographic string. Data from the motor vehicle administration are used to supplement identity linkage information. A combination of deterministic and probabilistic record linkage (Han & Lahiri, 2019; Scheuren & Winkler, 1993) is used depending on the amount of source information available for each record. If the record matches a current identity in the system, the MLDS Person ID is assigned, and the record is linked. A new identity with a new MLDS Person ID is created if the record does not match a current identity in the system. State agency staff deidentify the data and provide the deidentified data to researchers on a secured virtual server.

Maryland is a small mid-Atlantic state with 24 school districts, roughly defined by county borders. There are 23 counties in Maryland and Baltimore City is a separate school district. The state includes rural, town, suburban, and urban districts and schools. In 2021, there were 786 elementary schools, 213 middle schools, and 182 high schools in the state (MSDE, 2022a). As of fall 2021, Maryland public school students were 34% White, 33% Black, 21% Hispanic, 5% two or more races, and 7% Asian or other races (MSDE, 2022a). In 2021, 47% of students were eligible for free/reduced price meals, and this percentage ranged from 22% in Carroll County to 89% in Somerset County; several districts have high rates due to the Community Eligibility Provision, where free/reduced price meals are provided for large proportions of students by default (MSDE, 2021). Based on data from the National Assessment of Education Progress (NAEP) in 2019, Maryland scored similarly to the nation in terms of the percentage of students at or above proficient in 4th grade math and reading and in 8th grade math (U.S. Department of Education, Institute of Education Sciences, National Center for Education Statistics, 2019). The 2019 NAEP also indicated that Maryland scored significantly higher than the nation in terms of the percentage of students at or above proficient in 8th grade reading (36% in Maryland compared to 32% national). In 2021, Maryland’s 4-year adjusted cohort high school graduation rate was 87% (MSDE, 2022b). The gap in the 4-year adjusted cohort rate for Black and White students was 9 percentage points, and the Hispanic-White gap was 21 percentage points (MSDE, 2022b).

MHEC is responsible for the planning, supervision, and coordination of Maryland’s postsecondary education system. Four-year public institutions include the University System of Maryland (USM), which includes 11 institutions, including the flagship University of Maryland College Park and three Historically Black Colleges/Universities (HBCUs). There are two 4-year public institutions not included in USM: Morgan State University, a HBCU located in Baltimore City, and St. Mary’s College of Maryland, the national public honors college on the Eastern Shore of the state. There are 16 community colleges located across Maryland, and there are 13 state-aided independent institutions, including the Johns Hopkins University, which is the largest of these institutions. On average between 2007–2008 and 2019–2020, 49% of high school graduates enrolled in a full-time degree-seeking program in the fall after high school graduation (MLDS Center, 2022a). Sixty-six percent of these students earned a degree by the age of 25. About a third of students who enroll in college enroll in Maryland community colleges, about a third enroll in out-of-state institutions, just under a third enroll in Maryland public 4-year institutions, and about 5% enroll in Maryland state-aided independent institutions (MLDS Center, 2022b).

### Cohorts

Using the data defined above, we calculated attrition rates for five cohorts, defined using student enrollment data. Descriptive statistics for each cohort are provided in the supplemental materials. Analyses for elementary, middle, and high school were restricted to students attending at least 1 day of school in schools that served the full grade span examined in this study (i.e., K-5 for elementary school; 6–8 for middle school; and 9–12 for high school). Students attending schools with expanded or reduced grade ranges, special education schools, charter schools, or alternative schools at baseline were excluded from the study. Ninety percent of students in Maryland public schools attend general education schools that serve the full grade spans typically associated with elementary, middle, and high school in the USA.

#### Elementary School (ES)

The elementary school cohort included all kindergarten students enrolled in a public elementary school in the 2011–2012 academic year (*N* = 51,000^1^). The ES cohort was followed for 6 years, from kindergarten through fifth grade for students with a typical grade progression.

#### Middle School (MS)

The middle school cohort included all sixth-grade students enrolled in a public middle school in the 2013–2014 academic year (*N* = 52,000). The MS cohort was followed for 3 years, from sixth through eighth grade for students with a typical grade progression.

#### High School (HS) 

The high school cohort included all ninth-grade students enrolled in a public high school in the 2013–2014 academic year (*N* = 70,000). The HS cohort was followed for 4 years, from ninth through twelfth grade for students with a typical grade progression.

#### Postsecondary (High School Graduates; PS-HSG)

The PS-HSG cohort included all students who graduated from a public high school in the 2012–2013 academic year and enrolled in any Maryland college in fall 2013 as first-time degree-seeking undergraduates. Analyses were run separately for students who initially enrolled in an associate degree program (*N* = 13,000) and students who initially enrolled in a bachelor’s degree program (*N* = 10,000).

#### Postsecondary (Maryland College Enrollees; PS-MD)

The PS-MD cohort included all first-time degree-seeking undergraduates in Maryland colleges in fall 2013. Analyses were run separately for students initially enrolled in an associate degree program (*N* = 23,000) and students who were initially enrolled in a bachelor’s degree program (*N* = 20,000). The PS-HSG and the PS-MD cohorts were followed for 150% of time necessary to attain a degree. The associate degree is typically a 2-year degree, so students initially enrolled in an associate degree program were followed for 3 years. The bachelor’s degree is typically a 4-year degree, so students initially enrolled in a bachelor’s degree program were followed for 6 years.

### Defining Attrition

#### ES, MS, and HS Cohorts

Attrition was defined using a unique school identifier for each Maryland public school enrollment. The baseline (first school attended in the first cohort year) identifier was compared to subsequent school enrollments to classify students into four mutually exclusive groups in each academic year (see Rickles et al., 2018). For the ES, MS, and HS cohorts, students could (a) remain in the same school (no attrition); (b) move to a different school within the same school district (attrite from school); (c) move to a different school in a different district (attrite from district); or (d) leave the Maryland public school system (attrite from state). An additional grouping was added for the HS cohort to indicate completion of high school, which was not classified as attrition. Each academic year was examined using data from the entire school year. Attrition over the summer was included in the prior year’s attrition count (i.e., attrition over the summer between kindergarten and first grade was included in the attrition count for kindergarten). School attrition in the final year of each grade span (i.e., fifth, eighth, and twelfth grades) was not calculated because students are expected to change schools at the end of these grades (i.e., structural mobility). For all cohorts and years, attrition from the state was measured by examining attendance records in the first enrollment of the following academic year. If students did not have an enrollment record in a Maryland public school in the following academic year, the student was classified as attriting from the state.

#### PS-HSG and PS-MD Cohorts

A unique college identifier was used for each college enrollment in Maryland and for college enrollments in out-of-state institutions (for the PS-HSG cohort only). The baseline identifier (first fall enrollment) was compared to subsequent college enrollments to classify students into mutually exclusive groups in each academic year. For the PS-MD cohort, there were five mutually exclusive groups: (a) remain in the same college (no attrition); (b) move to a different college within the same state higher education system (i.e., 4-year public, community college, attrite from college); (c) move to a different college in a different higher education system^2^ (attrite from system); (d) leave the Maryland higher education system (attrite from state); or (e) obtain the initial degree sought, which was not classified as attrition. For the PS-HSG cohort, NSC data were available, and an additional category differentiated students who left the state and enrolled in an out-of-state postsecondary institution (attrite from state) and students who were no longer enrolled in any PS institution in Maryland or out-of-state (attrite from PS). Each academic year was examined using data from the entire school year (i.e., fall, winter, and spring). For both cohorts, attrition over the summer was included in the prior year’s attrition count (i.e., attrition over the summer between the first and second year of college was included in the attrition count for the first year).

### Measures

#### Student-Level Covariates

Student-level covariates that are typically used in school-based prevention studies and were available in the Maryland data were selected for inclusion in this study. Student-level covariates were measured in the year of initial enrollment and included (1) gender (male or female); (2) race/ethnicity (Hispanic of any race; non-Hispanic Black; non-Hispanic White; non-Hispanic Asian; or other); and for the ES, MS, and HS cohorts, (3) a binary variable indicating whether the student was eligible for free/reduced price meals (FRPL); (4) a binary variable indicating whether the student received English Language Learner (ELL) services; and (5) a binary variable indicating whether the student received special education (SPECED) services.

#### School-Level Covariates

School-level covariates for elementary, middle, and high schools were measured in the year of initial enrollment using data from the Common Core of Data (CCD) and included (1) school-level composition for FRPL; and (2) urbanicity (city/suburban/town/rural). School-level FRPL was categorized as high (75th percentile and above), medium (74th–26th percentile), and low (25th percentile and below). For Maryland bachelor’s degree seekers, attrition was examined by the initial postsecondary system of enrollment (i.e., 4-year state-aided; 4-year public; St. Mary’s; Morgan State).

#### Outcomes

The outcomes described were measured for the MS cohort because the timing for this cohort allowed for the analysis of test scores at baseline (prior to attrition measurement) and test score outcomes, along with high school graduation, postsecondary, and workforce outcomes. *Academic performance* was measured in fifth and eighth grade. Fifth grade academic performance was measured using students’ reading and math scale scores on the Maryland School Assessment (MSA; scale score range: 240 to 650; proficiency cutoff: 384 for reading and 392 for math), Maryland’s annual standardized assessment, aligning with the voluntary state curriculum at the time the MS cohort was in fifth grade. Eighth grade academic performance was measured using students’ scale scores on the Partnership for Assessment of Readiness for College and Career (PARCC; scale score range: 650 to 850) math and English Language Arts/Literacy (ELA/L) assessments (cutoff for meeting expectations: 750 for math and ELA/L). PARCC was Maryland’s state standardized test, aligning with the voluntary state curriculum when the MS cohort was in eighth grade. Students who took the Algebra I test in eighth grade did not take the PARCC math test and were not included in the analysis predicting math test scores. *High school graduation* (yes/no) was indicated when a student graduated from high school in any year up until 2020, the most recent year of data available when the analyses were conducted and the year of expected graduation for a typically progressing student in the MS cohort. *Postsecondary enrollment* (yes/no) was indicated when a student enrolled in any postsecondary institution after the year of on-time high school graduation for a typically progressing student (i.e., until 2021, the most recent year of data available when analyses were conducted). *Workforce participation (yes/no) and earnings* were measured for students who were not enrolled in a Maryland college by summing quarterly wage data from the Maryland UI records corresponding to academic years 2020–2021. Earnings were log transformed due to skewness.

### Analyses

#### Attrition Rates

Cumulative attrition rates were calculated by dividing the total number of students who attrited up until year *X* by the total number of students in the cohort. Cumulative attrition rates by student- and school-level covariates were calculated by dividing the total number of students in the subgroup who attrited up until year *X* by the total number of students in the subgroup in the cohort. Annual attrition rates provided in the supplemental materials were calculated by dividing the total number of students who attrited in year *X* by the total number of students in the cohort who were still enrolled (i.e., had not attrited in previous years) in year *X*. A similar method was used to calculate annual attrition rates by student- and school-level covariates, but the numerators and denominators included only students in the subgroup. Grouped bar charts were created to display cumulative attrition rates for each cohort and facilitate comparisons across student- and school-level covariates. Annual and cumulative attrition rates by year are provided for each cohort in the supplemental materials.

#### Outcome Analyses

To examine the association between attrition and student outcomes, linear regression was used for continuous outcomes (i.e., academic performance, log earnings) and logistic regression was used for binary outcomes (i.e., high school graduation, postsecondary enrollment, workforce participation). The main predictor of interest—attrition—is indicated when students attrited from either their school or district at any point during the middle school years (i.e., sixth through eighth grade). Outcomes were not observed for students who left the state, and these students were not included in the outcome analyses.

Outcome analyses were conducted conditional on fifth grade academic performance, as measured by standardized test scores. As such, the sample for the outcome analyses was restricted to students with a present fifth grade test score on either reading or math. We provide descriptive statistics for the sample used for the outcome analyses in the supplemental materials. Fifth grade math and reading scores were highly correlated (*r* = 0.71), so the two scores were averaged to form one single covariate to avoid multicollinearity. When a student only had one score present, that score was used. All continuous measures were standardized with a mean of 0 and a SD of 1. For continuous outcomes, the coefficient estimate for attrition represents the amount of a standard deviation change in the outcome for students who attrited compared to students who did not attrite.

## Results

### Cumulative Attrition Rates by Cohort

**Fig. 1:**
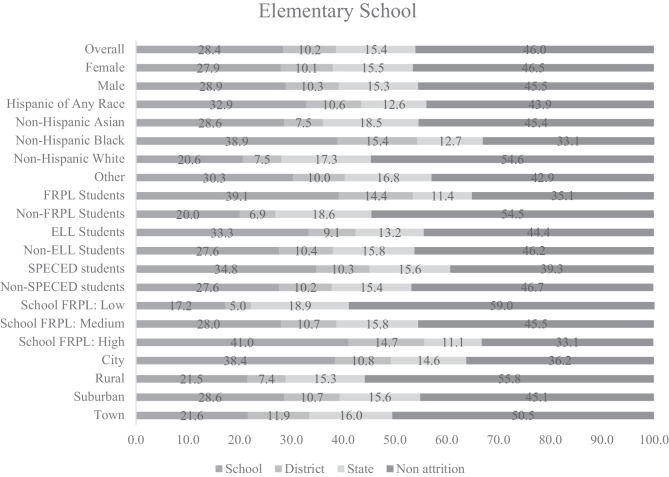
Cumulative student attrition rates for the elementary school cohort. No attrition = student remained in the same school; attrite from school = student moved to a different school within the same district; attrite from district = student moved to a different school in a different district; attrite from state = student is no longer found in the Maryland public school system. FRPL, eligibility for free/reduced price meals; ELL, English language learner; SPECED, special education.

**Fig. 2:**
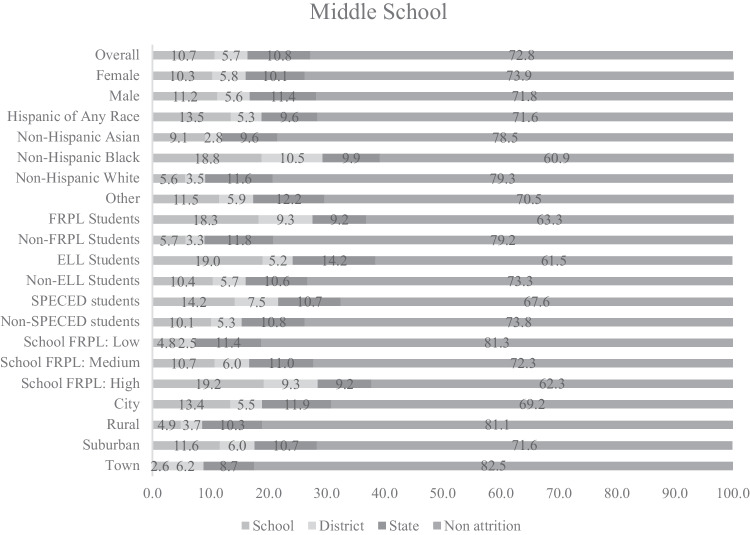
Cumulative student attrition rates for the middle school cohort. No attrition = student remained in the same school; attrite from school = student moved to a different school within the same district; attrite from district = student moved to a different school in a different district; attrite from state = student is no longer found in the Maryland public school system. FRPL, eligibility for free/reduced price meals; ELL, English language learner; SPECED, special education

**Fig. 3:**
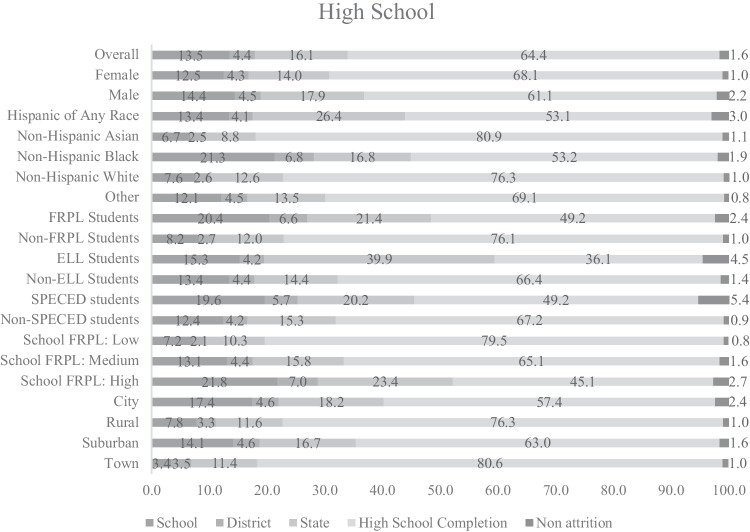
Cumulative student attrition rates for the high school cohort. No attrition = student remained in the same school; attrite from school = student moved to a different school within the same district; attrite from district = student moved to a different school in a different district; attrite from state = student is no longer found in the Maryland public school system. FRPL, eligibility for free/reduced price meals; ELL, English language learner; SPECED, special education.

Cumulative attrition rates are displayed in Figs. 1, 2, and 3. By the end of fifth grade, 54% of students in the ES cohort attrited from their first elementary school attended (28% attrited from school but stayed in district; 10% attrited from district but stayed in state; and 15% attrited from state; see Fig. 1). By the end of eighth grade, 27% of students in the MS cohort attrited from their first middle school attended (11% attrited from school; 6% attrited from district; and 11% attrited from state; see Fig. 2). By the end of twelfth grade, 34% of students in the HS cohort attrited from their first high school attended (14% attrited from school; 4% attrited from district; and 16% attrited from state; see Fig. 3). Cumulative attrition rates for the PS-MD and PS-HSG cohorts are provided in the supplemental materials and were calculated at 150% of the time necessary to earn a degree. We followed the cohort of associate degree seekers for 3 years and of bachelor’s degree seekers for 6 years. After 3 years of college, 73% of associate degree seekers in the PS-MD cohort attrited from their first college attended (5% attrited from college but stayed in system; 13% attrited from system but stayed in state; and 55% attrited from the state but may have enrolled in college out-of-state). After 6 years of college, 45% of bachelor’s degree seekers in the PS-MD cohort attrited from their first college attended (4% attrited from college; 19% attrited from system; and 22% attrited from the state but may have enrolled in college out-of-state). After 3 years of college, 68% of associate degree seekers in the PS-HSG cohort attrited from their first college attended (5% attrited from college but stayed in system; 16% attrited from system but stayed in state; 6% attrited from the state postsecondary system; and 41% attrited from postsecondary all together [determined using data from the NSC]). After 6 years of college, 50% of bachelor’s degree seekers in the PS-HSG cohort attrited from their first college attended (5% attrited from college; 28% attrited from system; 3% attrited from the state postsecondary system; and 13% attrited from postsecondary all together [determined using data from the NSC]).

### Cumulative Attrition Rates by Student-Level Covariates

Non-Hispanic Black students had the highest rates of attrition for the ES, MS, and HS cohorts (see Figs. 1–3). Hispanic students of any race had the second highest rates of attrition. For the ES, MS, and HS cohorts, students who were eligible for FRPL had higher rates of attrition than students who were not eligible for FRPL, ELL students had higher rates of attrition than students who were non-ELL (although this difference is less pronounced in HS when compared to MS and ES), and SPECED students had higher rates of attrition than students who were non-SPECED. Male students had higher rates of attrition for the HS cohort only. Non-Hispanic Black students had the highest rates of attrition in the PS-HSG and PS-MD cohorts for associate and bachelor’s degree seekers. Cumulative and annual attrition rates for each student-level covariate are provided by year and attrition category in the supplemental materials.

### Cumulative Attrition Rates by School-Level Covariates

For the ES, MS, and HS cohorts, students attending schools with high levels of students eligible for FRPL had higher rates of attrition than students attending schools with medium or low levels (see Figs. 1–3). Students attending urban schools had higher rates of attrition than students attending rural, suburban, and town schools. Students attending suburban schools had the second highest rates of attrition and only differed from urban schools by about two and a half percentage points in MS. Cumulative and annual attrition rates for each school-level covariate are provided by year and attrition category in the supplemental materials. Additionally, the supplemental materials include cumulative and annual attrition rates for the PS-HSG and PS-MD bachelor’s degree cohorts by the type of postsecondary system of initial enrollment.

### Outcome Analysis

The results for the outcome analysis are displayed in Table 1. Conditional on fifth grade academic performance, attrition in middle school was significantly associated with lower eighth grade reading (*b* =  −0.16, *p* < .0001) and math (*b* =  −0.21, *p* < .0001) scores, lower rates of high school graduation (*b* =  −0.54, *p* < .0001) and college enrollment (*b* =  −0.22, *p* < .0001), and lower log earnings after high school (*b* =  −0.06, *p* < .0001). Attrition was not significantly related to workforce participation after controlling for fifth grade academic performance.

**Table 1:** Standardized regression results of the outcome analyses conditional on fifth grade academic performance

	*b*	*SE*	*p*
Continuous outcomes			
8th grade ELA			
Intercept	0.01	0.00	0.02
Attrition	−0.16	0.01	< .0001
5th grade test score	0.74	0.00	< .0001
8th grade math			
Intercept	0.02	0.00	< .0001
Attrition	−0.21	0.01	< .0001
5th grade test score	0.75	0.00	< .0001
Log earnings			
Intercept	−0.02	0.01	0.05
Attrition	−0.06	0.02	0.002
5th grade test score	−0.17	0.01	< .0001
Binary outcomes			
High school graduation			
Intercept	1.81	0.02	< .0001
Attrition	−0.54	0.02	< .0001
5th grade test score	0.62	0.02	< .0001
PS enrollment			
Intercept	−.46	0.01	< .0001
Attrition	−0.22	0.01	< .0001
5th grade test score	0.36	0.01	< .0001
Workforce participation			
Intercept	0.38	0.02	< .0001
Attrition	−0.03	0.02	0.12
5th grade test score	−0.06	0.01	< .0001

All continuous measures were standardized with a mean of 0 and a SD of 1. For continuous outcomes, the coefficient estimate for attrition represents the amount of a standard deviation change in the outcome for students who attrited compared to students who did not attrite

*ELA* English language arts

## Discussion

Attrition is a critical concern for researchers planning and conducting school-based prevention studies. The WWC and the EEF, initiatives concerned with determining the quality of school-based interventions in the USA and the UK, respectively, emphasize the importance of attrition because it may lead to problems with internal validity. The current study provides rates of attrition for a state population and subgroups of students and schools who are often sampled by prevention scientists. This is the first study to provide practical guidance for expected rates of attrition using population-level state data; our findings can help researchers to proactively plan for specific levels of attrition in the study design phase, limiting bias and increasing study validity.

Our results suggest that researchers using K-12 samples should plan for attrition rates as high as 27% during middle school and 54% during elementary school but should consider the grade levels sampled (see annual attrition rates in the supplemental materials), the specific characteristics of the students in the sample of interest, and the specific schools available to be sampled. Researchers using postsecondary samples should plan for attrition rates as high as 73% for associate degree seekers and 45% for bachelor’s degree seekers. Because attrition can also result from other sources (e.g., nonresponse; drop out after randomization), the attrition rates we provide should be used as a lower bound for estimating actual attrition. Additionally, we leveraged the unique nature of using statewide data to observe outcomes for students who attrited from their school or district but stayed in the state public school system and compare them to students who never attrited. We found that attrition in middle school was associated with lower eighth grade academic performance, lower rates of high school graduation and postsecondary enrollment, and lower log earnings after high school, even after controlling for fifth grade academic performance, indicating that attrition is likely to be nonignorable.

The empirical attrition rates provided in this study are specific to one state-level population, and researchers using these rates as guidance should be particularly mindful of how the demographics and context of their specific studies match the demographics and context of Maryland’s student population. Maryland’s public education system includes 24 school districts that roughly align with county borders and Baltimore City borders. In other states, districts are typically much smaller in geographic size and larger in quantity across the state. The estimates of district attrition may be smaller in Maryland when compared to other states because of the large geographic size of districts and the change in urbanicity, political atmosphere, and/or population demographics that may accompany movement to a new county in the state. Similarly, the estimates of school attrition may be larger in Maryland because students may be moving to different schools that are within the same district in Maryland but would be in different districts in a state with smaller district geographical boundaries. Temporal and contextual factors, such as local economic or housing conditions, may also influence attrition rates and should be considered when estimating attrition rates for specific evaluation studies.

The postsecondary attrition rates we report for the population of students who begin in Maryland postsecondary institutions (PS-MD cohort) are specific to the state of Maryland and do not include enrollments for students in out-of-state institutions. Postsecondary institutions typically have access to extensive data on their student populations (e.g., financial aid information; support program participation; course taking) that can supplement the information that is typically available in statewide data and national administrative datasets like the NSC. We also provide attrition rates for the population of high school students who enroll in a Maryland postsecondary institution (PS-HSG cohort), a cohort for whom we had access to NSC data through the State Department of Education. Examining attrition with this cohort enabled us to differentiate between enrolling in an out-of-state institution (i.e., left Maryland but still in college) and leaving PS all together (i.e., left Maryland and not found in an out-of-state institution due to dropping out or entering the workforce). By comparing the findings from the PS-MD cohort and the PS-HSG cohort, we can infer that a large proportion of the students leaving Maryland’s postsecondary system are leaving college all together, rather than enrolling in out-of-state institutions.

The outcome analyses examining the association between attrition in middle school and long-term outcomes indicated that attrition is a concern that is nonignorable when examining the long-run impacts of prevention policies and programs. After controlling for fifth grade standardized test scores, students who attrited from their school or school district had eighth grade ELA and math scores 0.16 and 0.21 standard deviations lower than students who did not attrite. Our model predicts that a student who scored 1 standard deviation above the mean in fifth grade will score 0.75 standard deviations above the mean in eighth grade ELA and 0.77 standard deviations above the mean in eighth grade math, assuming no attrition. Contrast this with predicted eighth grade ELA and math scores 0.59 and 0.56 standard deviations above the mean for students who experienced attrition; it is evident that attrition has noticeable impacts on test scores. Furthermore, we see even greater impacts for high school graduation. For students who did not attrite, a student who scored 1 standard deviation above the mean in fifth grade had a predicted probability of 0.92 of graduating, but a student with the same fifth grade test score who experienced attrition had a predicted graduation probability of 0.86. These differences increase for students scoring below the mean. For example, for students who scored two standard deviations below the mean, the predicted graduation probability was 0.64 for students who did not attrite but dropped to 0.51 for students with attrition.

The expected attrition rates we provide can be used by researchers to plan for attrition, and our results suggest that specific subgroups of students and schools may need to be oversampled to meet the requirements for the MDES. Our results suggest that Hispanic students, non-Hispanic Black students, ELL students, SPECED students, and schools with high concentrations of students who are eligible for free/reduced price meals may need to be oversampled in K-12. High rates of K-12 attrition were found in elementary and middle school, suggesting a particular importance of oversampling when researchers are interested in these developmental periods. Postsecondary attrition rates were the highest for non-Hispanic Black students and associate degree seekers, suggesting a need for oversampling when engaging with these postsecondary populations.

Additionally, researchers can use our results to determine cost-effective data sources for follow-up. Researchers conducting studies with samples expected to have low attrition rates might opt for primary data collection, whereas researchers conducting studies with samples expected to have high attrition rates might opt for using administrative data, or, if primary data collection is needed for the evaluation, develop a sampling design that would be less vulnerable to attrition. Our findings comparing attrition rates from school, district, and the state can help to guide researchers on partnerships to obtain administrative data for follow-up. For example, our findings show the highest rates of attrition are to a different school within the same district for elementary school students, suggesting that district partnerships might be sufficient for longitudinal follow-up. For middle and high school students, the rates of attrition from school and the state public school system were similar, indicating potential benefits from additional data sources outside of state longitudinal data systems for follow-up. Attrition rates for postsecondary samples were high, and results indicate a particular need for oversampling associate degree seekers, who had the highest rates of leaving postsecondary all together.

## Conclusion

The prevention and education fields are increasingly calling for follow-up studies to examine the long-run impacts of programs and policies (e.g., Watts et al., 2019), as evidenced by recent additions to the Institute of Education Sciences request for applications for efficacy studies focused on long-term follow-up. However, attrition becomes an increasingly problematic concern as studies follow students over time and becomes particularly problematic when attrition leads to bias in the effect estimates for outcomes. The findings from the current study can be used to estimate plausible values of attrition during the study design phase to help limit future problems with internal validity. Furthermore, these findings can be used to determine cost effective data sources (i.e., districtwide data, statewide data, national data) for longitudinal follow-up studies.

## Supplementary Information

Below is the link to the electronic supplementary material.

Supplementary file1 (DOCX 216 KB)

## Data Availability

The MLDS data are protected under state and federal privacy laws and are not available publicly. The processed data sets and code can be made available to individuals who complete the MLDS Center’s access authorization process (see mldscenter.maryland.gov).

## Footnotes

1 *N*s were rounded to the nearest thousand to comply with Maryland’s suppression guidelines which follow the Federal Education Rights and Privacy Act (FERPA) guidelines provided by the National Center for Education Statistics.

2 Students who left Morgan State University and St. Mary’s College of Maryland were classified into the *attrite from system* group.
