# Supplementary material for: Estimating Student Attrition in School-Based Prevention Studies: Guidance from State Longitudinal Data in Maryland
Source: Prev Sci. 2023 May 17;24(5):1035–45. doi: 10.1007/s11121-023-01533-1 (PMC10409654; doi:10.1007/s11121-023-01533-1)
Supplement: Supplementary file 1 — Supplementary file1 (DOCX 216 KB) [file 11121_2023_1533_MOESM1_ESM.docx]

**Estimating Student Attrition in School-Based Prevention Studies: Guidance from State Longitudinal Data in Maryland**

**(Supplemental Materials)**

Angela K. Henneberger, University of Maryland School of Social Work

[ahenneberger@ssw.umaryland.edu](mailto:ahenneberger@ssw.umaryland.edu)

Bess A. Rose, University of Maryland School of Social Work

Yi Feng, University of Maryland College Park

Tessa Johnson, University of Maryland College Park

Brennan Register, University of Maryland College Park

Laura M. Stapleton, University of Maryland College Park

Tracy Sweet, University of Maryland College Park

Michael Woolley, University of Maryland School of Social Work

Acknowledgements: The authors would like to thank Yating Zheng and Alison Preston for assistance with preparing this manuscript. We are grateful for the data, technical, and research support provided by the Maryland Longitudinal Data System (MLDS) Center and its agency partners. The views and opinions expressed are those of the authors and do not necessarily represent the views of the MLDS Center or its agency partners. The research reported here was developed under a grant from the Department of Education Institute of Education Sciences (IES SLDS Grant: R372A150045). However, these contents do not necessarily represent the policy of the Department of Education, and you should not assume endorsement by the Federal Government.

Supplemental Table 1. *Cumulative Attrition for the ES Cohort (N = 51,000)*

|  |  |  | **Year 1** | **Year 2** | **Year 3** | **Year 4** | **Year 5** | **Year 6** |
| --- | --- | --- | --- | --- | --- | --- | --- | --- |
| Total |  | Non attrition | 80.6 | 69.4 | 60.6 | 53.9 | 49.4 | 46.0 |
|  |  | School | 10.3 | 16.5 | 21.7 | 25.7 | 28.2 | 28.4 |
|  |  | District | 4.3 | 6.2 | 7.5 | 8.6 | 9.3 | 10.2 |
|  |  | State | 4.9 | 7.9 | 10.2 | 11.8 | 13.2 | 15.4 |
| Gender | Female  (N=25,000) | Non attrition | 80.7 | 69.7 | 61.0 | 54.4 | 49.8 | 46.5 |
|  |  | School | 10.1 | 16.2 | 21.2 | 25.2 | 27.6 | 27.9 |
|  |  | District | 4.2 | 6.0 | 7.5 | 8.5 | 9.3 | 10.1 |
|  |  | State | 5.0 | 8.1 | 10.3 | 11.9 | 13.3 | 15.5 |
|  | Male  (N=27,000) | Non attrition | 80.5 | 69.1 | 60.2 | 53.4 | 48.9 | 45.5 |
|  |  | School | 10.4 | 16.8 | 22.2 | 26.3 | 28.7 | 28.9 |
|  |  | District | 4.4 | 6.3 | 7.6 | 8.6 | 9.3 | 10.3 |
|  |  | State | 4.7 | 7.8 | 10.1 | 11.7 | 13.0 | 15.3 |
| Race/Ethnicity | Hispanic of Any Race  (N=7,000) | Non attrition | 80.0 | 68.3 | 58.8 | 51.6 | 46.5 | 43.9 |
|  |  | School | 11.2 | 18.4 | 24.9 | 29.6 | 32.6 | 32.9 |
|  |  | District | 4.3 | 6.3 | 7.7 | 8.8 | 9.8 | 10.6 |
|  |  | State | 4.4 | 6.9 | 8.6 | 10.0 | 11.1 | 12.6 |
|  | Non-Hispanic Asian  (N=4,000) | Non attrition | 84.7 | 71.2 | 61.6 | 52.6 | 48.5 | 45.4 |
|  |  | School | 7.3 | 14.6 | 20.1 | 26.2 | 28.4 | 28.6 |
|  |  | District | 2.3 | 3.7 | 4.9 | 6.0 | 6.4 | 7.5 |
|  |  | State | 5.7 | 10.5 | 13.4 | 15.2 | 16.7 | 18.5 |
|  | Non-Hispanic Black  (N=14,000) | Non attrition | 72.7 | 58.7 | 48.4 | 40.9 | 36.2 | 33.1 |
|  |  | School | 15.6 | 24.2 | 31.0 | 35.8 | 38.6 | 38.9 |
|  |  | District | 6.9 | 9.8 | 11.7 | 13.1 | 14.1 | 15.4 |
|  |  | State | 4.7 | 7.3 | 9.0 | 10.1 | 11.1 | 12.7 |
|  | Non-Hispanic White  (N=24,000) | Non attrition | 84.9 | 75.8 | 68.4 | 62.7 | 58.4 | 54.6 |
|  |  | School | 7.3 | 11.7 | 15.5 | 18.4 | 20.5 | 20.6 |
|  |  | District | 2.9 | 4.4 | 5.4 | 6.3 | 6.8 | 7.5 |
|  |  | State | 4.8 | 8.2 | 10.6 | 12.6 | 14.3 | 17.3 |
|  | Other  (N=3,000) | Non attrition | 79.1 | 68.1 | 57.7 | 50.6 | 46.4 | 42.9 |
|  |  | School | 10.6 | 17.0 | 23.3 | 27.6 | 30.1 | 30.3 |
|  |  | District | 4.7 | 6.6 | 7.7 | 8.7 | 9.2 | 10.0 |
|  |  | State | 5.7 | 8.3 | 11.3 | 13.2 | 14.2 | 16.8 |
| FRPL (Student) | FRPL Students  (N=23,000) | Non attrition | 73.7 | 60.2 | 49.9 | 42.6 | 37.6 | 35.1 |
|  |  | School | 15.4 | 23.8 | 30.8 | 35.6 | 38.8 | 39.1 |
|  |  | District | 6.4 | 9.2 | 11.0 | 12.3 | 13.3 | 14.4 |
|  |  | State | 4.4 | 6.8 | 8.3 | 9.5 | 10.3 | 11.4 |
|  | Non-FRPL Students  (N=29,000) | Non attrition | 86.0 | 76.6 | 69.0 | 62.7 | 58.6 | 54.5 |
|  |  | School | 6.2 | 10.8 | 14.6 | 18.0 | 19.9 | 20.0 |
|  |  | District | 2.6 | 3.8 | 4.8 | 5.7 | 6.2 | 6.9 |
|  |  | State | 5.2 | 8.8 | 11.6 | 13.6 | 15.4 | 18.6 |
| ELL | ELL Students  (N=7,000) | Non attrition | 81.4 | 69.6 | 59.6 | 51.9 | 46.7 | 44.4 |
|  |  | School | 10.7 | 17.8 | 24.5 | 29.7 | 32.9 | 33.3 |
|  |  | District | 3.3 | 5.0 | 6.3 | 7.3 | 8.3 | 9.1 |
|  |  | State | 4.5 | 7.6 | 9.6 | 11.1 | 12.1 | 13.2 |
|  | Non-ELL Students  (N=45,000) | Non attrition | 80.5 | 69.4 | 60.8 | 54.2 | 49.8 | 46.2 |
|  |  | School | 10.2 | 16.3 | 21.3 | 25.1 | 27.4 | 27.6 |
|  |  | District | 4.4 | 6.4 | 7.7 | 8.8 | 9.5 | 10.4 |
|  |  | State | 4.9 | 8.0 | 10.2 | 11.9 | 13.3 | 15.8 |
| SPECED | SPECED students  (N=5,000) | Non attrition | 76.0 | 63.3 | 53.5 | 47.3 | 42.8 | 39.3 |
|  |  | School | 15.2 | 22.3 | 28.6 | 32.4 | 34.7 | 34.8 |
|  |  | District | 4.5 | 6.8 | 8.0 | 8.9 | 9.5 | 10.3 |
|  |  | State | 4.3 | 7.6 | 9.9 | 11.4 | 13.0 | 15.6 |
|  | Non-SPECED students  (N=46,000) | Non attrition | 81.1 | 70.1 | 61.4 | 54.6 | 50.1 | 46.7 |
|  |  | School | 9.7 | 15.8 | 21.0 | 25.0 | 27.4 | 27.6 |
|  |  | District | 4.2 | 6.1 | 7.5 | 8.5 | 9.3 | 10.2 |
|  |  | State | 4.9 | 8.0 | 10.2 | 11.9 | 13.2 | 15.4 |
| FRPL (School) | School FRPL: Low  (N=13,000) | Non attrition | 87.9 | 79.2 | 72.3 | 66.6 | 63.3 | 59.0 |
|  |  | School | 5.4 | 9.5 | 13.0 | 16.0 | 17.1 | 17.2 |
|  |  | District | 2.0 | 3.0 | 3.6 | 4.1 | 4.5 | 5.0 |
|  |  | State | 4.7 | 8.4 | 11.1 | 13.3 | 15.1 | 18.9 |
|  | School FRPL: Medium  (N=25,000) | Non attrition | 80.2 | 69.1 | 60.0 | 53.2 | 48.8 | 45.5 |
|  |  | School | 10.1 | 16.2 | 21.4 | 25.4 | 27.8 | 28.0 |
|  |  | District | 4.4 | 6.4 | 7.9 | 9.0 | 9.7 | 10.7 |
|  |  | State | 5.2 | 8.3 | 10.7 | 12.4 | 13.7 | 15.8 |
|  | School FRPL: High  (N=13,000) | Non attrition | 73.5 | 59.5 | 49.2 | 41.5 | 35.8 | 33.1 |
|  |  | School | 15.8 | 24.6 | 31.6 | 36.9 | 40.7 | 41.0 |
|  |  | District | 6.3 | 9.2 | 11.1 | 12.5 | 13.5 | 14.7 |
|  |  | State | 4.4 | 6.7 | 8.0 | 9.1 | 10.0 | 11.1 |
| Urbanicity | City  (N=7,000) | Non attrition | 75.5 | 62.5 | 53.0 | 45.5 | 39.9 | 36.2 |
|  |  | School | 16.1 | 23.7 | 29.9 | 34.9 | 38.1 | 38.4 |
|  |  | District | 3.9 | 6.5 | 7.9 | 9.0 | 9.7 | 10.8 |
|  |  | State | 4.5 | 7.3 | 9.2 | 10.7 | 12.2 | 14.6 |
|  | Rural  (N=9,000) | Non attrition | 84.7 | 76.7 | 69.4 | 63.6 | 58.7 | 55.8 |
|  |  | School | 7.6 | 11.3 | 15.4 | 18.6 | 21.3 | 21.5 |
|  |  | District | 3.3 | 4.6 | 5.5 | 6.3 | 6.8 | 7.4 |
|  |  | State | 4.4 | 7.4 | 9.7 | 11.5 | 13.1 | 15.3 |
|  | Suburban  (N=32,000) | Non attrition | 80.5 | 68.7 | 59.6 | 52.8 | 48.6 | 45.1 |
|  |  | School | 9.8 | 16.7 | 22.1 | 26.2 | 28.4 | 28.6 |
|  |  | District | 4.5 | 6.5 | 7.9 | 9.0 | 9.8 | 10.7 |
|  |  | State | 5.1 | 8.1 | 10.4 | 12.0 | 13.3 | 15.6 |
|  | Town  (N=3,000) | Non attrition | 81.1 | 71.5 | 62.8 | 56.6 | 53.0 | 50.5 |
|  |  | School | 8.5 | 12.2 | 16.8 | 19.9 | 21.5 | 21.6 |
|  |  | District | 5.3 | 7.4 | 8.8 | 9.9 | 10.8 | 11.9 |
|  |  | State | 5.1 | 8.9 | 11.5 | 13.5 | 14.6 | 16.0 |

*Notes.* FRPL = eligibility for free/reduced price meals; ELL = English language learner; SPECED = special education.

Supplemental Table 2. *Cumulative Attrition for the MS Cohort (N =52,000)*

|  |  |  | **Year 1** | **Year 2** | **Year 3** |
| --- | --- | --- | --- | --- | --- |
| Total |  | Non attrition | 86.1 | 78.9 | 72.8 |
|  |  | School | 6.9 | 10.2 | 10.7 |
|  |  | District | 3.0 | 4.4 | 5.7 |
|  |  | State | 4.0 | 6.5 | 10.8 |
| Gender | Female  (N=25,000) | Non attrition | 86.6 | 79.4 | 73.9 |
|  |  | School | 6.5 | 9.8 | 10.3 |
|  |  | District | 3.1 | 4.5 | 5.8 |
|  |  | State | 3.9 | 6.3 | 10.1 |
|  | Male  (N=27,000) | Non attrition | 85.6 | 78.4 | 71.8 |
|  |  | School | 7.3 | 10.6 | 11.2 |
|  |  | District | 3.0 | 4.3 | 5.6 |
|  |  | State | 4.1 | 6.7 | 11.4 |
| Race/Ethnicity | Hispanic of Any Race  (N=7,000) | Non attrition | 84.7 | 76.6 | 71.6 |
|  |  | School | 8.7 | 12.7 | 13.5 |
|  |  | District | 2.6 | 4.0 | 5.3 |
|  |  | State | 4.0 | 6.6 | 9.6 |
|  | Non-Hispanic Asian  (N=4,000) | Non attrition | 88.4 | 82.4 | 78.5 |
|  |  | School | 6.2 | 9.0 | 9.1 |
|  |  | District | 1.4 | 2.0 | 2.8 |
|  |  | State | 4.0 | 6.7 | 9.6 |
|  | Non-Hispanic Black  (N=14,000) | Non attrition | 78.2 | 67.5 | 60.9 |
|  |  | School | 11.8 | 17.8 | 18.8 |
|  |  | District | 6.0 | 8.4 | 10.5 |
|  |  | State | 3.9 | 6.3 | 9.9 |
|  | Non-Hispanic White  (N=25,000) | Non attrition | 90.6 | 85.5 | 79.3 |
|  |  | School | 3.7 | 5.4 | 5.6 |
|  |  | District | 1.8 | 2.6 | 3.5 |
|  |  | State | 4.0 | 6.5 | 11.6 |
|  | Other  (N=3,000) | Non attrition | 85.6 | 77.3 | 70.5 |
|  |  | School | 7.0 | 10.8 | 11.5 |
|  |  | District | 2.7 | 4.6 | 5.9 |
|  |  | State | 4.6 | 7.3 | 12.2 |
| FRPL (Student) | FRPL Students  (N=21,000) | Non attrition | 79.3 | 68.8 | 63.3 |
|  |  | School | 11.4 | 17.3 | 18.3 |
|  |  | District | 5.1 | 7.3 | 9.3 |
|  |  | State | 4.1 | 6.6 | 9.2 |
|  | Non-FRPL Students  (N=31,000) | Non attrition | 90.6 | 85.6 | 79.2 |
|  |  | School | 3.8 | 5.5 | 5.7 |
|  |  | District | 1.7 | 2.5 | 3.3 |
|  |  | State | 3.9 | 6.4 | 11.8 |
| ELL | ELL Students  (N=2,000) | Non attrition | 79.9 | 66.8 | 61.5 |
|  |  | School | 10.7 | 17.9 | 19.0 |
|  |  | District | 2.5 | 4.0 | 5.2 |
|  |  | State | 6.9 | 11.3 | 14.2 |
|  | Non-ELL Students  (N=50,000) | Non attrition | 86.3 | 79.4 | 73.3 |
|  |  | School | 6.7 | 9.9 | 10.4 |
|  |  | District | 3.1 | 4.4 | 5.7 |
|  |  | State | 3.9 | 6.3 | 10.6 |
| SPECED | SPECED students  (N=8,000) | Non attrition | 82.6 | 73.4 | 67.6 |
|  |  | School | 8.9 | 13.5 | 14.2 |
|  |  | District | 4.1 | 5.8 | 7.5 |
|  |  | State | 4.3 | 7.3 | 10.7 |
|  | Non-SPECED students  (N=44,000) | Non attrition | 86.7 | 79.9 | 73.8 |
|  |  | School | 6.5 | 9.6 | 10.1 |
|  |  | District | 2.8 | 4.1 | 5.3 |
|  |  | State | 4.0 | 6.4 | 10.8 |
| FRPL (School) | School FRPL: Low  (N=15,000) | Non attrition | 92.1 | 87.6 | 81.3 |
|  |  | School | 3.1 | 4.6 | 4.8 |
|  |  | District | 1.4 | 2.0 | 2.5 |
|  |  | State | 3.5 | 5.8 | 11.4 |
|  | School FRPL: Medium  (N=27,000) | Non attrition | 85.7 | 78.3 | 72.3 |
|  |  | School | 6.9 | 10.2 | 10.7 |
|  |  | District | 3.1 | 4.6 | 6.0 |
|  |  | State | 4.3 | 6.9 | 11.0 |
|  | School FRPL: High  (N=10,000) | Non attrition | 78.7 | 68.2 | 62.3 |
|  |  | School | 12.0 | 18.2 | 19.2 |
|  |  | District | 5.2 | 7.3 | 9.3 |
|  |  | State | 4.1 | 6.4 | 9.2 |
| Urbanicity | City  (N=9,000) | Non attrition | 83.9 | 75.2 | 69.2 |
|  |  | School | 8.4 | 12.9 | 13.4 |
|  |  | District | 3.0 | 4.3 | 5.5 |
|  |  | State | 4.6 | 7.6 | 11.9 |
|  | Rural  (N=6,000) | Non attrition | 91.1 | 85.9 | 81.1 |
|  |  | School | 2.8 | 4.8 | 4.9 |
|  |  | District | 1.8 | 2.9 | 3.7 |
|  |  | State | 4.2 | 6.4 | 10.3 |
|  | Suburban  (N=34,000) | Non attrition | 85.3 | 78.0 | 71.6 |
|  |  | School | 7.5 | 11.0 | 11.6 |
|  |  | District | 3.2 | 4.7 | 6.0 |
|  |  | State | 3.9 | 6.3 | 10.7 |
|  | Town  (N=2,000) | Non attrition | 92.2 | 87.5 | 82.5 |
|  |  | School | 1.6 | 2.5 | 2.6 |
|  |  | District | 3.3 | 4.6 | 6.2 |
|  |  | State | 3.0 | 5.4 | 8.7 |

*Notes.* FRPL = eligibility for free/reduced price meals; ELL = English language learner; SPECED = special education.

Supplemental Table 3. *Cumulative Attrition for the HS Cohort (N =70,000)*

|  |  |  | **Year 1** | **Year 2** | **Year 3** | **Year 4** |
| --- | --- | --- | --- | --- | --- | --- |
| Total |  | Non attrition | 82.1 | 72.6 | 65.0 | 1.6 |
|  |  | School | 7.8 | 11.5 | 13.1 | 13.5 |
|  |  | District | 2.8 | 3.9 | 4.4 | 4.4 |
|  |  | State | 7.4 | 11.7 | 14.5 | 16.1 |
|  |  | High School Completion | 0.0 | 0.3 | 3.0 | 64.4 |
| Gender | Female  (N=33,000) | Non attrition | 83.4 | 74.7 | 67.6 | 1.0 |
|  |  | School | 7.2 | 10.8 | 12.2 | 12.5 |
|  |  | District | 2.7 | 3.8 | 4.3 | 4.3 |
|  |  | State | 6.7 | 10.4 | 12.8 | 14.0 |
|  |  | High School Completion | 0.0 | 0.3 | 3.1 | 68.1 |
|  | Male  (N=37,000) | Non attrition | 80.9 | 70.8 | 62.7 | 2.2 |
|  |  | School | 8.2 | 12.1 | 14.0 | 14.4 |
|  |  | District | 2.8 | 3.9 | 4.4 | 4.5 |
|  |  | State | 8.1 | 12.9 | 16.1 | 17.9 |
|  |  | High School Completion | 0.0 | 0.3 | 2.8 | 61.1 |
| Race/Ethnicity | Hispanic of Any Race  (N=11,000) | Non attrition | 79.5 | 66.6 | 55.0 | 3.0 |
|  |  | School | 7.4 | 11.2 | 12.8 | 13.4 |
|  |  | District | 2.3 | 3.6 | 4.0 | 4.1 |
|  |  | State | 10.8 | 18.3 | 23.6 | 26.4 |
|  |  | High School Completion | 0.0 | 0.4 | 4.6 | 53.1 |
|  | Non-Hispanic Asian  (N=4,000) | Non attrition | 89.5 | 84.2 | 79.9 | 1.1 |
|  |  | School | 4.5 | 6.0 | 6.6 | 6.7 |
|  |  | District | 1.6 | 2.1 | 2.5 | 2.5 |
|  |  | State | 4.5 | 7.2 | 8.4 | 8.8 |
|  |  | High School Completion | 0.0 | 0.4 | 2.6 | 80.9 |
|  | Non-Hispanic Black  (N=25,000) | Non attrition | 73.3 | 62.1 | 53.3 | 1.9 |
|  |  | School | 13.2 | 18.4 | 20.8 | 21.3 |
|  |  | District | 4.5 | 6.2 | 6.8 | 6.8 |
|  |  | State | 9.0 | 13.0 | 15.4 | 16.8 |
|  |  | High School Completion | 0.0 | 0.4 | 3.7 | 53.2 |
|  | Non-Hispanic White  (N=27,000) | Non attrition | 89.7 | 82.7 | 77.1 | 1.0 |
|  |  | School | 3.5 | 6.2 | 7.4 | 7.6 |
|  |  | District | 1.5 | 2.2 | 2.5 | 2.6 |
|  |  | State | 5.3 | 8.9 | 11.3 | 12.6 |
|  |  | High School Completion | 0.0 | 0.1 | 1.7 | 76.3 |
|  | Other  (N=3,000) | Non attrition | 84.9 | >75 | 68.9 | 0.8 |
|  |  | School | 7.3 | 10.8 | 11.9 | 12.1 |
|  |  | District | 2.8 | 3.8 | 4.5 | 4.5 |
|  |  | State | 5.1 | 9.5 | 11.9 | 13.5 |
|  |  | High School Completion | 0.0 | <0.4 | 2.8 | 69.1 |
| FRPL (Student) | FRPL Students  (N=30,000) | Non attrition | 74.4 | 61.1 | 50.6 | 2.4 |
|  |  | School | 12.0 | 17.5 | 19.9 | 20.4 |
|  |  | District | 4.1 | 5.8 | 6.5 | 6.6 |
|  |  | State | 9.5 | 15.2 | 19.0 | 21.4 |
|  |  | High School Completion | 0.0 | 0.4 | 4.0 | 49.2 |
|  | Non-FRPL Students  (N=39,000) | Non attrition | 87.9 | 81.5 | 76.0 | 1.0 |
|  |  | School | 4.5 | 6.8 | 8.0 | 8.2 |
|  |  | District | 1.8 | 2.4 | 2.7 | 2.7 |
|  |  | State | 5.8 | 9.1 | 11.1 | 12.0 |
|  |  | High School Completion | 0.0 | 0.2 | 2.2 | 76.1 |
| ELL | ELL Students  (N=5,000) | Non attrition | 73.1 | 54.6 | 39.2 | 4.5 |
|  |  | School | 8.9 | 13.4 | 14.7 | 15.3 |
|  |  | District | 2.4 | 3.7 | 4.2 | 4.2 |
|  |  | State | 15.6 | 27.3 | 35.7 | 39.9 |
|  |  | High School Completion | 0.0 | 0.9 | 6.2 | 36.1 |
|  | Non-ELL Students  (N=65,000) | Non attrition | 82.7 | 73.9 | 66.8 | 1.4 |
|  |  | School | 7.7 | 11.3 | 13.0 | 13.4 |
|  |  | District | 2.8 | 3.9 | 4.4 | 4.4 |
|  |  | State | 6.8 | 10.7 | 13.1 | 14.4 |
|  |  | High School Completion | 0.0 | 0.2 | 2.7 | 66.4 |
| SPECED | SPECED students  (N=11,000) | Non attrition | 75.4 | 63.7 | 54.8 | 5.4 |
|  |  | School | 11.9 | 16.7 | 18.8 | 19.6 |
|  |  | District | 3.7 | 5.0 | 5.6 | 5.7 |
|  |  | State | 9.1 | 14.3 | 17.7 | 20.2 |
|  |  | High School Completion | 0.0 | 0.3 | 3.1 | 49.2 |
|  | Non-SPECED students  (N=59,000) | Non attrition | 83.3 | 74.3 | 66.9 | 0.9 |
|  |  | School | 7.0 | 10.5 | 12.1 | 12.4 |
|  |  | District | 2.6 | 3.7 | 4.1 | 4.2 |
|  |  | State | 7.1 | 11.3 | 14.0 | 15.3 |
|  |  | High School Completion | 0.0 | 0.3 | 2.9 | 67.2 |
| FRPL (School) | School FRPL: Low  (N=17,000) | Non attrition | 89.6 | 84.6 | 79.5 | 0.8 |
|  |  | School | 4.3 | 5.7 | 7.0 | 7.2 |
|  |  | District | 1.4 | 1.9 | 2.1 | 2.1 |
|  |  | State | 4.7 | 7.7 | 9.6 | 10.3 |
|  |  | High School Completion | 0.0 | 0.0 | 0.4 | 79.5 |
|  | School FRPL: Medium  (N=38,000) | Non attrition | 82.9 | 72.9 | 65.3 | 1.6 |
|  |  | School | 7.4 | 11.4 | 12.8 | 13.1 |
|  |  | District | 2.7 | 3.9 | 4.4 | 4.4 |
|  |  | State | 7.0 | 11.4 | 14.3 | 15.8 |
|  |  | High School Completion | 0.0 | 0.3 | 3.2 | 65.1 |
|  | School FRPL: High  (N=14,000) | Non attrition | 71.3 | 57.9 | 47.4 | 2.7 |
|  |  | School | 12.6 | 18.2 | 21.0 | 21.8 |
|  |  | District | 4.6 | 6.2 | 6.9 | 7.0 |
|  |  | State | 11.5 | 17.3 | 21.0 | 23.4 |
|  |  | High School Completion | 0.0 | 0.4 | 3.7 | 45.1 |
| Urbanicity | City  (N=15,000) | Non attrition | 78.1 | 66.9 | 60.0 | 2.4 |
|  |  | School | 10.2 | 15.4 | 16.9 | 17.4 |
|  |  | District | 2.9 | 4.0 | 4.6 | 4.6 |
|  |  | State | 8.8 | 13.4 | 16.3 | 18.2 |
|  |  | High School Completion | 0.0 | 0.2 | 2.1 | 57.4 |
|  | Rural  (N=11,000) | Non attrition | 87.9 | 81.6 | 76.7 | 1.0 |
|  |  | School | 5.0 | 7.0 | 7.6 | 7.8 |
|  |  | District | 2.1 | 2.9 | 3.3 | 3.3 |
|  |  | State | 5.0 | 8.3 | 10.4 | 11.6 |
|  |  | High School Completion | 0.0 | 0.1 | 2.1 | 76.3 |
|  | Suburban  (N=42,000) | Non attrition | 81.4 | 71.6 | 62.9 | 1.6 |
|  |  | School | 7.9 | 11.7 | 13.7 | 14.1 |
|  |  | District | 3.0 | 4.1 | 4.6 | 4.6 |
|  |  | State | 7.7 | 12.3 | 15.2 | 16.7 |
|  |  | High School Completion | 0.0 | 0.3 | 3.6 | 63.0 |
|  | Town  (N=2,000) | Non attrition | 92.3 | 87.2 | 81.9 | 1.0 |
|  |  | School | 1.9 | 2.7 | 3.3 | 3.4 |
|  |  | District | 2.1 | 3.0 | 3.5 | 3.5 |
|  |  | State | 3.8 | 7.0 | 9.9 | 11.4 |
|  |  | High School Completion | 0.0 | 0.0 | 1.4 | 80.6 |

*Notes.* FRPL = eligibility for free/reduced price meals; ELL = English language learner; SPECED = special education.

Supplemental Table 4. *Cumulative Attrition for the PS-HSG Associate Degree Cohort (N =13,000)*

|  | |  | **Year 1** | **Year 2** | **Year 3** |
| --- | --- | --- | --- | --- | --- |
| Total |  | Obtained degree | 0.2 | 4.9 | 13.1 |
|  |  | No Attrition | 66.4 | 41.0 | 18.8 |
|  |  | College | 2.9 | 4.1 | 4.9 |
|  |  | System | 5.9 | 11.4 | 16.1 |
|  |  | State | 3.1 | 5.0 | 5.9 |
|  |  | Postsecondary | 21.6 | 33.6 | 41.3 |
| Gender | Female  (N=7,000) | Obtained degree | 0.2 | 5.3 | 13.9 |
|  |  | No Attrition | 67.6 | 41.6 | 19.1 |
|  |  | College | 3.0 | 4.5 | 5.4 |
|  |  | System | 6.0 | 11.2 | 15.5 |
|  |  | State | 3.2 | 5.4 | 6.3 |
|  |  | Postsecondary | 20.0 | 32.0 | 39.7 |
|  | Male  (N=6,000) | Obtained degree | 0.2 | 4.4 | 12.2 |
|  |  | No Attrition | 65.0 | 40.4 | 18.4 |
|  |  | College | 2.9 | 3.7 | 4.3 |
|  |  | System | 5.7 | 11.5 | 16.6 |
|  |  | State | 3.0 | 4.6 | 5.4 |
|  |  | Postsecondary | 23.3 | 35.3 | 43.1 |
| Race/Ethnicity | Hispanic of Any Race  (N=1,000) | Obtained degree | <1 | 3.7 | 12.4 |
|  |  | No Attrition | 76.5 | 53.8 | 28.3 |
|  |  | College | 2.7 | 3.8 | 4.3 |
|  |  | System | 2.9 | 8.0 | 14.0 |
|  |  | State | <3 | 3.8 | 4.1 |
|  |  | Postsecondary | 15.6 | 26.9 | 36.9 |
|  | Non-Hispanic Asian  (N=1,000) | Obtained degree | 0.0 | 6.6 | 16.7 |
|  |  | No Attrition | 80.4 | 54.5 | 25.7 |
|  |  | College | 1.4 | 1.8 | 2.8 |
|  |  | System | 8.9 | 18.3 | 28.8 |
|  |  | State | 1.8 | 3.2 | 3.7 |
|  |  | Postsecondary | 7.6 | 15.7 | 22.4 |
|  | Non-Hispanic Black  (N=4,000) | Obtained degree | <1 | 1.3 | 4.7 |
|  |  | No Attrition | 57.6 | 34.4 | 16.1 |
|  |  | College | 4.2 | 5.8 | 6.9 |
|  |  | System | 5.2 | 9.2 | 13.3 |
|  |  | State | <3 | 4.4 | 5.1 |
|  |  | Postsecondary | 30.1 | 44.9 | 53.9 |
|  | Non-Hispanic White  (N=6,000) | Obtained degree | 0.2 | 7.3 | 18.1 |
|  |  | No Attrition | 68.0 | 40.5 | 17.5 |
|  |  | College | 2.3 | 3.2 | 3.9 |
|  |  | System | 6.4 | 12.6 | 16.3 |
|  |  | State | 3.6 | 5.9 | 7.0 |
|  |  | Postsecondary | 19.5 | 30.5 | 37.2 |
|  | Other  (N=1,000) | Obtained degree | <1 | 5.2 | 15.3 |
|  |  | No Attrition | 67.5 | 44.5 | 19.5 |
|  |  | College | 3.4 | 4.8 | 5.4 |
|  |  | System | 7.6 | 12.9 | 19.3 |
|  |  | State | <4 | 5.1 | 6.1 |
|  |  | Postsecondary | 18.0 | 27.5 | 34.4 |

Supplemental Table 5. *Cumulative Attrition for the PS-HSG Bachelor’s Degree Cohort (N =10,000)*

|  | |  | **Year 1** | **Year 2** | **Year 3** | **Year 4** | **Year 5** | **Year 6** |
| --- | --- | --- | --- | --- | --- | --- | --- | --- |
| Total |  | Obtained degree | 0.0 | 0.0 | 0.7 | 35.4 | 47.3 | 49.4 |
|  |  | No Attrition | 75.0 | 65.0 | 57.4 | 17.8 | 4.0 | 1.1 |
|  |  | College | 2.1 | 3.4 | 4.3 | 4.6 | 4.7 | 4.7 |
|  |  | System | 17.9 | 22.9 | 26.6 | 27.9 | 28.1 | 28.2 |
|  |  | State | 1.5 | 2.2 | 2.8 | 3.1 | 3.1 | 3.1 |
|  |  | Postsecondary | 3.6 | 6.4 | 8.2 | 11.3 | 12.8 | 13.4 |
| Gender | Female  (n=6,000) | Obtained degree | 0.0 | 0.0 | 0.8 | 37.7 | 48.4 | 50.2 |
|  |  | No Attrition | 74.4 | 64.2 | 56.2 | 15.2 | 3.1 | 0.9 |
|  |  | College | 2.0 | 3.5 | 4.3 | 4.6 | 4.7 | 4.7 |
|  |  | System | 19.0 | 24.3 | 28.6 | 29.8 | 29.9 | 30.0 |
|  |  | State | 1.6 | 2.4 | 3.1 | 3.4 | 3.4 | 3.4 |
|  |  | Postsecondary | 3.0 | 5.6 | 7.0 | 9.3 | 10.4 | 10.9 |
|  | Male  (n=5,000) | Obtained degree | 0.0 | <1 | 0.7 | 32.4 | 45.9 | 48.5 |
|  |  | No Attrition | 75.8 | 66.0 | 58.8 | 21.0 | 5.0 | 1.4 |
|  |  | College | 2.1 | 3.4 | 4.2 | 4.6 | 4.8 | 4.8 |
|  |  | System | 16.5 | 21.1 | 24.2 | 25.6 | 25.9 | 25.9 |
|  |  | State | 1.3 | <3 | 2.4 | 2.7 | 2.7 | 2.7 |
|  |  | Postsecondary | 4.3 | 7.4 | 9.7 | 13.7 | 15.8 | 16.7 |
| Race/Ethnicity | Hispanic of Any Race  (n=1000) | Obtained degree | 0.0 | 0.0 | <1 | 32.0 | 46.6 | 49.3 |
|  |  | No Attrition | 73.5 | 66.1 | 58.2 | 20.9 | 3.9 | <1 |
|  |  | College | 1.1 | 2.7 | 3.1 | 3.5 | 3.5 | <4 |
|  |  | System | 20.4 | 23.5 | 27.5 | 28.9 | 29.2 | 29.2 |
|  |  | State | 1.6 | 2.2 | <3 | 3.1 | 3.1 | <4 |
|  |  | Postsecondary | 3.5 | 5.5 | 7.7 | 11.6 | 13.7 | 14.3 |
|  | Non-Hispanic Asian  (n=1000) | Obtained degree | <1 | <1 | 1.1 | 42.9 | 54.3 | 56.2 |
|  |  | No Attrition | 76.0 | 68.8 | 62.1 | 16.2 | 3.1 | <1 |
|  |  | College | 2.9 | 4.2 | 5.4 | 5.7 | 5.8 | 5.9 |
|  |  | System | 19.2 | 22.3 | 25.4 | 26.7 | 26.8 | 26.8 |
|  |  | State | <1 | <2 | 2.0 | 2.1 | 2.1 | <3 |
|  |  | Postsecondary | <1 | 3.1 | 4.0 | 6.4 | 7.8 | 8.2 |
|  | Non-Hispanic Black  (n=3000) | Obtained degree | 0.0 | 0.0 | <1 | 20.8 | 34.9 | 38.3 |
|  |  | No Attrition | 74.1 | 59.6 | 50.9 | 23.7 | 6.7 | 2.0 |
|  |  | College | 2.3 | 4.3 | 4.9 | 5.4 | 5.6 | 5.6 |
|  |  | System | 15.6 | 22.2 | 26.3 | 28.5 | 28.8 | 28.9 |
|  |  | State | 1.5 | 2.6 | <4 | 3.6 | 3.7 | 3.7 |
|  |  | Postsecondary | 6.5 | 11.3 | 14.5 | 18.1 | 20.4 | 21.5 |
|  | Non-Hispanic White  (n=5000) | Obtained degree | 0.0 | 0.0 | 1.1 | 43.2 | 53.6 | 54.9 |
|  |  | No Attrition | 75.5 | 67.2 | 60.2 | 14.1 | 2.4 | 0.7 |
|  |  | College | 1.9 | 2.8 | 3.8 | 4.0 | 4.1 | 4.1 |
|  |  | System | 18.3 | 23.2 | 26.6 | 27.3 | 27.5 | 27.5 |
|  |  | State | 1.6 | 2.3 | 2.8 | 3.0 | 3.0 | 3.0 |
|  |  | Postsecondary | 2.6 | 4.5 | 5.6 | 8.3 | 9.4 | 9.8 |
|  | Other  (n=500) | Obtained degree | 0.0 | 0.0 | <2 | 36.8 | 46.7 | 49.2 |
|  |  | No Attrition | 75.3 | 66.3 | 57.6 | 15.4 | 4.1 | <2 |
|  |  | College | <2 | <4 | 3.5 | 4.1 | 4.1 | 4.1 |
|  |  | System | 21.0 | 26.1 | 30.7 | 32.1 | 32.3 | 32.5 |
|  |  | State | <2 | <2 | <3 | 2.5 | 2.5 | <3 |
|  |  | Postsecondary | <2 | <4 | 5.1 | 9.1 | 10.3 | 10.5 |
| Postsecondary System | 4-Year State-Aided  (MICUA; n=1500) | Obtained degree | 0.0 | 0.0 | 0.7 | 42.2 | 46.7 | 46.9 |
|  |  | No Attrition | 71.7 | 59.9 | 51.0 | 6.2 | 0.7 | <1 |
|  |  | College | <1 | 0.7 | 1.0 | 1.0 | 1.0 | <1 |
|  |  | System | 22.1 | 30.4 | 36.4 | 37.6 | 37.7 | 37.8 |
|  |  | State | <3 | 3.4 | 4.4 | 4.5 | 4.5 | 4.5 |
|  |  | Postsecondary | 3.3 | 5.6 | 6.5 | 8.6 | 9.4 | 9.7 |
|  | Morgan  (n=500) | Obtained degree | 0.0 | 0.0 | 0.0 | 12.7 | 30.7 | 35.1 |
|  |  | No Attrition | 78.2 | 62.2 | 52.3 | 33.2 | 11.0 | 4.6 |
|  |  | College | N/A | N/A | N/A | N/A | N/A | N/A |
|  |  | System | 11.8 | 20.5 | 25.3 | 27.8 | 28.2 | 28.6 |
|  |  | State | <2 | 3.3 | 4.6 | 4.8 | 4.8 | 4.8 |
|  |  | Postsecondary | <10 | 13.9 | 17.8 | 21.6 | 25.3 | 27.0 |
|  | St. Mary’s (n=300) | Obtained degree | 0.0 | 0.0 | <4 | 51.8 | 56.5 | 56.9 |
|  |  | No Attrition | 77.5 | 68.0 | 58.9 | <6 | <4 | 0.0 |
|  |  | College | N/A | N/A | N/A | N/A | N/A | N/A |
|  |  | System | 19.8 | 26.9 | 34.0 | 35.2 | 35.6 | 35.6 |
|  |  | State | <4 | <4 | <4 | <4 | <4 | <4 |
|  |  | Postsecondary | <4 | <4 | <4 | <6 | 5.1 | <6 |
|  | 4-Year Public (USM; n=8000) | Obtained degree | 0.0 | 0.0 | 0.8 | 35.0 | 48.1 | 50.5 |
|  |  | No Attrition | 75.3 | 66.0 | 58.8 | 19.3 | 4.2 | 1.1 |
|  |  | College | 2.5 | 4.2 | 5.3 | 5.7 | 5.8 | 5.9 |
|  |  | System | 17.4 | 21.6 | 24.7 | 25.9 | 26.1 | 26.1 |
|  |  | State | 1.3 | 2.0 | 2.4 | 2.7 | 2.7 | 2.8 |
|  |  | Postsecondary | 3.4 | 6.1 | 8.1 | 11.3 | 12.9 | 13.6 |

Supplemental Table 6. *Cumulative Attrition for the PS-MD Associate Degree Cohort (N =23,000)*

|  | |  | **Year 1** | **Year 2** | **Year 3** |
| --- | --- | --- | --- | --- | --- |
| Total |  | Obtained degree | 0.3 | 3.7 | 10.3 |
|  |  | No Attrition | 59.0 | 36.3 | 17.2 |
|  |  | College | 2.9 | 3.9 | 4.6 |
|  |  | System | 5.1 | 9.2 | 12.9 |
|  |  | State | 32.7 | 46.9 | 55.0 |
| Gender | Female  (n=12,000) | Obtained degree | 0.3 | 3.9 | 10.8 |
|  |  | No Attrition | 60.8 | 37.8 | 18.5 |
|  |  | College | 3.0 | 4.1 | 4.9 |
|  |  | System | 5.2 | 9.0 | 12.5 |
|  |  | State | 30.7 | 45.1 | 53.3 |
|  | Male  (n=11,000) | Obtained degree | 0.3 | 3.5 | 9.7 |
|  |  | No Attrition | 57.2 | 34.7 | 15.8 |
|  |  | College | 2.8 | 3.6 | 4.2 |
|  |  | System | 5.1 | 9.4 | 13.4 |
|  |  | State | 34.6 | 48.8 | 56.8 |
| Race/Ethnicity | Hispanic of Any Race  (n=2,000) | Obtained degree | <0.6 | 3.1 | 10.3 |
|  |  | No Attrition | 67.2 | 45.7 | 23.2 |
|  |  | College | <3 | 3.4 | 4.1 |
|  |  | System | 3.2 | 6.9 | 11.6 |
|  |  | State | 26.8 | 40.8 | 50.8 |
|  | Non-Hispanic Asian  (n=1,000) | Obtained degree | <0.2 | 5.0 | 14.4 |
|  |  | No Attrition | 73.6 | 50.0 | 23.5 |
|  |  | College | <2 | 2.4 | 3.5 |
|  |  | System | 7.2 | 14.8 | 23.1 |
|  |  | State | 17.1 | 27.8 | 35.5 |
|  | Non-Hispanic Black  (n=8,000) | Obtained degree | 0.2 | 1.1 | 3.9 |
|  |  | No Attrition | 51.6 | 30.8 | 15.5 |
|  |  | College | 3.8 | 5.0 | 5.8 |
|  |  | System | 4.4 | 7.2 | 10.6 |
|  |  | State | 40.1 | 55.9 | 64.1 |
|  | Non-Hispanic White  (11,000) | Obtained degree | 0.4 | 5.8 | 14.5 |
|  |  | No Attrition | 61.4 | 36.7 | 16.5 |
|  |  | College | 2.3 | 3.2 | 3.8 |
|  |  | System | 5.8 | 10.4 | 13.6 |
|  |  | State | 30.1 | 43.9 | 51.6 |
|  | Other  (n=1,000) | Obtained degree | <2 | 4.2 | 12.9 |
|  |  | No Attrition | 62.7 | 41.1 | 17.6 |
|  |  | College | <4 | 4.3 | 5.4 |
|  |  | System | 6.1 | 10.7 | 15.9 |
|  |  | State | 27.9 | 39.8 | 48.2 |

*Notes. N* = 36 students were missing gender information; *N* = 60 students were missing race/ethnicity information.

Supplemental Table 7. *Cumulative Attrition for the PS-MD Bachelor’s Degree Cohort (N =20,000)*

|  | |  | **Year 1** | **Year 2** | **Year 3** | **Year 4** | **Year 5** | **Year 6** |
| --- | --- | --- | --- | --- | --- | --- | --- | --- |
| Total |  | Obtained degree | 0.0 | 0.0 | 1.0 | 43.2 | 53.0 | 54.5 |
|  |  | No Attrition | 77.1 | 67.8 | 61.2 | 14.4 | 2.9 | 0.8 |
|  |  | College | 1.5 | 2.5 | 3.2 | 3.4 | 3.5 | 3.5 |
|  |  | System | 11.7 | 15.4 | 18.1 | 19.0 | 19.1 | 19.2 |
|  |  | State | 9.7 | 14.3 | 16.6 | 20.0 | 21.4 | 22.0 |
| Gender | Female  (n=11,000) | Obtained degree | 0.0 | 0.0 | 1.1 | 45.9 | 54.5 | 55.7 |
|  |  | No Attrition | 76.8 | 67.5 | 60.9 | 12.3 | 2.3 | 0.7 |
|  |  | College | 1.4 | 2.6 | 3.3 | 3.5 | 3.6 | 3.6 |
|  |  | System | 12.3 | 16.2 | 19.2 | 20.0 | 20.1 | 20.1 |
|  |  | State | 9.5 | 13.8 | 15.6 | 18.4 | 19.5 | 19.9 |
|  | Male  (n=9,000) | Obtained degree | 0.0 | 0.0 | 0.8 | 40.0 | 51.3 | 53.1 |
|  |  | No Attrition | 77.6 | 68.2 | 61.6 | 17.1 | 3.7 | 1.0 |
|  |  | College | 1.5 | 2.4 | 3.1 | 3.3 | 3.4 | 3.5 |
|  |  | System | 11.1 | 14.4 | 16.8 | 17.8 | 18.0 | 18.0 |
|  |  | State | 9.8 | 14.9 | 17.7 | 21.9 | 23.6 | 24.4 |
| Race/Ethnicity | Hispanic of Any Race  (n=1,000) | Obtained degree | 0.0 | 0.0 | 1.1 | 43.8 | 54.0 | 55.6 |
|  |  | No Attrition | 77.5 | 69.0 | 62.6 | 15.0 | 2.9 | 0.6 |
|  |  | College | 0.7 | 1.6 | 2.0 | 2.3 | 2.4 | 2.4 |
|  |  | System | 12.1 | 14.6 | 17.1 | 18.0 | 18.2 | 18.2 |
|  |  | State | 9.7 | 14.8 | 17.2 | 20.9 | 22.5 | 23.2 |
|  | Non-Hispanic Asian  (n=2,000) | Obtained degree | 0.0 | 0.0 | 1.8 | 50.5 | 61.5 | 62.9 |
|  |  | No Attrition | 81.0 | 74.5 | 67.4 | 15.1 | 2.6 | 0.7 |
|  |  | College | 2.0 | 3.1 | 4.0 | 4.2 | 4.2 | 4.3 |
|  |  | System | 13.5 | 16.4 | 19.2 | 20.1 | 20.2 | 20.2 |
|  |  | State | 3.4 | 6.0 | 7.6 | 10.1 | 11.4 | 11.9 |
|  | Non-Hispanic Black  (n=5,000) | Obtained degree | 0.0 | 0.0 | 0.5 | 23.0 | 35.4 | 38.2 |
|  |  | No Attrition | 70.7 | 57.0 | 49.1 | 21.0 | 5.7 | 1.7 |
|  |  | College | 1.9 | 3.6 | 4.2 | 4.6 | 4.8 | 4.8 |
|  |  | System | 12.4 | 17.6 | 20.7 | 22.3 | 22.7 | 22.8 |
|  |  | State | 14.9 | 21.7 | 25.4 | 29.1 | 31.4 | 32.5 |
|  | Non-Hispanic White  (n=10,000) | Obtained degree | 0.0 | 0.0 | 1.0 | 52.0 | 60.3 | 61.2 |
|  |  | No Attrition | 79.5 | 71.9 | 65.9 | 11.2 | 1.7 | 0.5 |
|  |  | College | 1.3 | 2.1 | 2.8 | 3.0 | 3.0 | 3.0 |
|  |  | System | 11.2 | 14.4 | 17.0 | 17.4 | 17.5 | 17.5 |
|  |  | State | 7.9 | 11.6 | 13.3 | 16.4 | 17.4 | 17.7 |
|  | Other  (n=1,000) | Obtained degree | 0.0 | 0.0 | 0.7 | 45.7 | 54.7 | 56.1 |
|  |  | No Attrition | 80.9 | 71.3 | 64.9 | 13.4 | 2.4 | 0.6 |
|  |  | College | 0.8 | 1.9 | 2.4 | 2.8 | 2.8 | 2.8 |
|  |  | System | 11.2 | 15.0 | 17.6 | 18.3 | 18.5 | 18.6 |
|  |  | State | 7.1 | 11.8 | 14.5 | 19.8 | 21.6 | 22.0 |
| Postsecondary System | 4-Year State -Aided (MICUA; n=6,000) | Obtained degree | 0.0 | 0.0 | 1.5 | 59.8 | 63.7 | 64.0 |
|  |  | No Attrition | 82.0 | 73.6 | 67.4 | 5.4 | 0.6 | 0.1 |
|  |  | College | 0.3 | 0.5 | 0.7 | 0.7 | 0.7 | 0.7 |
|  |  | System | 8.6 | 12.3 | 15.0 | 15.5 | 15.6 | 15.6 |
|  |  | State | 9.1 | 13.6 | 15.4 | 18.6 | 19.4 | 19.5 |
|  | Morgan  (n=900) | Obtained degree | 0.0 | 0.0 | 0.1 | 15.6 | 33.1 | 36.9 |
|  |  | No Attrition | 75.2 | 60.4 | 51.7 | 30.8 | 9.4 | 3.7 |
|  |  | College | N/A | N/A | N/A | N/A | N/A | N/A |
|  |  | System | 8.2 | 13.9 | 17.3 | 19.2 | 19.5 | 19.8 |
|  |  | State | 16.6 | 25.7 | 30.9 | 34.4 | 38.0 | 39.6 |
|  | St. Mary’s  (n=400) | Obtained degree | 0.0 | 0.0 | 0.8 | 53.4 | 57.7 | 57.9 |
|  |  | No Attrition | 76.7 | 68.8 | 61.1 | 5.8 | 0.8 | 0.3 |
|  |  | College | N/A | N/A | N/A | N/A | N/A | N/A |
|  |  | System | 19.6 | 25.4 | 31.2 | 32.3 | 32.5 | 32.5 |
|  |  | State | 3.7 | 5.8 | 6.9 | 8.5 | 9.0 | 9.3 |
|  | 4-Year Public (USM; n=13,000) | Obtained degree | 0.0 | 0.0 | 0.8 | 37.0 | 49.2 | 51.2 |
|  |  | No Attrition | 75.0 | 65.5 | 58.9 | 17.8 | 3.6 | 1.0 |
|  |  | College | 2.2 | 3.7 | 4.7 | 5.0 | 5.2 | 5.2 |
|  |  | System | 13.2 | 16.7 | 19.2 | 20.2 | 20.3 | 20.4 |
|  |  | State | 9.6 | 14.1 | 16.4 | 20.0 | 21.6 | 22.3 |

*Notes. N* = 11 students were missing gender information; *N* = 108 students were missing race/ethnicity information.

Supplemental Table 8. *Annual Attrition for the ES Cohort*

|  |  |  | **Year 1** | **Year 2** | **Year 3** | **Year 4** | **Year 5** | **Year 6** |
| --- | --- | --- | --- | --- | --- | --- | --- | --- |
|  |  |  | (N=51,000) | (N=41,000) | (N=36,000) | (N=31,000) | (N=28,000) | (N=25,000) |
| Total |  | Non attrition | 80.6 | 86.1 | 87.3 | 88.9 | 91.6 | 93.2 |
|  |  | School | 10.3 | 7.7 | 7.6 | 6.6 | 4.5 | 0.4 |
|  |  | District | 4.3 | 2.4 | 1.9 | 1.7 | 1.4 | 1.8 |
|  |  | State | 4.9 | 3.8 | 3.2 | 2.7 | 2.5 | 4.6 |
| Gender | Female  (N=25,000) | Non attrition | 80.7 | 86.4 | 87.6 | 89.1 | 91.7 | 93.3 |
|  |  | School | 10.1 | 7.5 | 7.3 | 6.4 | 4.5 | 0.5 |
|  |  | District | 4.2 | 2.3 | 2.0 | 1.8 | 1.3 | 1.8 |
|  |  | State | 5.0 | 3.8 | 3.1 | 2.7 | 2.5 | 4.5 |
|  | Male  (N=27,000) | Non attrition | 80.5 | 85.8 | 87.1 | 88.8 | 91.6 | 93.0 |
|  |  | School | 10.4 | 7.9 | 7.8 | 6.8 | 4.5 | 0.4 |
|  |  | District | 4.4 | 2.4 | 1.8 | 1.7 | 1.4 | 1.9 |
|  |  | State | 4.7 | 3.8 | 3.3 | 2.8 | 2.5 | 4.7 |
| Race/Ethnicity | Hispanic of Any Race  (N=7,000) | Non attrition | 80.0 | 85.4 | 86.0 | 87.8 | 90.1 | 94.5 |
|  |  | School | 11.2 | 9.0 | 9.5 | 8.0 | 5.9 | 0.7 |
|  |  | District | 4.3 | 2.5 | 2.0 | 1.8 | 2.0 | 1.7 |
|  |  | State | 4.4 | 3.1 | 2.5 | 2.4 | 2.1 | 3.1 |
|  | Non-Hispanic Asian  (N=4,000) | Non attrition | 84.7 | 84.0 | 86.5 | 85.3 | 92.3 | 93.6 |
|  |  | School | 7.3 | 8.6 | 7.7 | 10.0 | 4.1 | 0.4 |
|  |  | District | 2.3 | 1.7 | 1.6 | 1.7 | 0.8 | 2.3 |
|  |  | State | 5.7 | 5.6 | 4.1 | 3.0 | 2.8 | 3.7 |
|  | Non-Hispanic Black  (N=14,000) | Non attrition | 72.8 | 80.8 | 82.3 | 84.5 | 88.6 | 91.3 |
|  |  | School | 15.6 | 11.8 | 11.6 | 10.1 | 6.8 | 0.7 |
|  |  | District | 6.9 | 3.9 | 3.2 | 3.1 | 2.3 | 3.5 |
|  |  | State | 4.7 | 3.5 | 2.9 | 2.4 | 2.3 | 4.4 |
|  | Non-Hispanic White  (N=24,000) | Non attrition | 84.9 | 89.3 | 90.3 | 91.6 | 93.1 | 93.5 |
|  |  | School | 7.3 | 5.1 | 5.0 | 4.3 | 3.3 | 0.3 |
|  |  | District | 2.9 | 1.7 | 1.4 | 1.2 | 0.9 | 1.2 |
|  |  | State | 4.9 | 3.9 | 3.3 | 2.9 | 2.7 | 5.1 |
|  | Other  (N=3,000) | Non attrition | 79.1 | 86.2 | 84.7 | 87.7 | 91.8 | 92.4 |
|  |  | School | 10.6 | 8.1 | 9.3 | 7.3 | 5.1 | 0.4 |
|  |  | District | 4.7 | 2.4 | 1.6 | 1.7 | 1.1 | 1.6 |
|  |  | State | 5.7 | 3.4 | 4.3 | 3.3 | 2.1 | 5.6 |
| FRPL (Student) | FRPL Students  (N=23,000) | Non attrition | 73.7 | 81.6 | 82.9 | 85.5 | 88.2 | 93.4 |
|  |  | School | 15.4 | 11.4 | 11.6 | 9.6 | 7.5 | 0.9 |
|  |  | District | 6.4 | 3.8 | 3.0 | 2.6 | 2.3 | 3.0 |
|  |  | State | 4.4 | 3.2 | 2.5 | 2.3 | 2.0 | 2.8 |
|  | Non-FRPL Students  (N=29,000) | Non attrition | 86.0 | 89.1 | 90.0 | 90.9 | 93.5 | 93.0 |
|  |  | School | 6.2 | 5.3 | 5.1 | 4.9 | 2.9 | 0.2 |
|  |  | District | 2.6 | 1.4 | 1.3 | 1.2 | 0.9 | 1.2 |
|  |  | State | 5.2 | 4.2 | 3.6 | 3.0 | 2.7 | 5.6 |
| ELL | ELL Students  (N=7,000) | Non attrition | 81.4 | 85.6 | 85.5 | 87.2 | 89.9 | 95.1 |
|  |  | School | 10.7 | 8.6 | 9.7 | 8.6 | 6.2 | 0.8 |
|  |  | District | 3.3 | 2.0 | 2.0 | 1.7 | 1.9 | 1.8 |
|  |  | State | 4.6 | 3.8 | 2.8 | 2.5 | 2.0 | 2.3 |
|  | Non-ELL Students  (N=45,000) | Non attrition | 80.5 | 86.2 | 87.6 | 89.2 | 91.9 | 92.9 |
|  |  | School | 10.2 | 7.6 | 7.2 | 6.3 | 4.3 | 0.4 |
|  |  | District | 4.4 | 2.5 | 1.9 | 1.8 | 1.3 | 1.8 |
|  |  | State | 4.9 | 3.8 | 3.3 | 2.8 | 2.6 | 5.0 |
| SPECED | SPECED students  (N=5,000) | Non attrition | 76.0 | 83.4 | 84.5 | 88.4 | 90.4 | 91.8 |
|  |  | School | 15.2 | 9.3 | 9.9 | 7.1 | 4.9 | 0.4 |
|  |  | District | 4.5 | 3.0 | 1.9 | 1.7 | 1.3 | 1.8 |
|  |  | State | 4.3 | 4.4 | 3.7 | 2.8 | 3.4 | 6.1 |
|  | Non-SPECED students  (N=46,000) | Non attrition | 81.1 | 86.4 | 87.6 | 89.0 | 91.8 | 93.3 |
|  |  | School | 9.7 | 7.6 | 7.3 | 6.6 | 4.5 | 0.4 |
|  |  | District | 4.2 | 2.3 | 1.9 | 1.7 | 1.4 | 1.8 |
|  |  | State | 4.9 | 3.7 | 3.2 | 2.7 | 2.4 | 4.5 |
| FRPL (School) | School FRPL: Low  (N=13,000) | Non attrition | 87.9 | 90.1 | 91.4 | 92.1 | 95.0 | 93.2 |
|  |  | School | 5.4 | 4.7 | 4.5 | 4.1 | 1.7 | 0.2 |
|  |  | District | 2.0 | 1.1 | 0.8 | 0.8 | 0.6 | 0.7 |
|  |  | State | 4.7 | 4.2 | 3.4 | 3.0 | 2.7 | 6.0 |
|  | School FRPL: Medium  (N=25,000) | Non attrition | 80.2 | 86.2 | 86.8 | 88.7 | 91.6 | 93.4 |
|  |  | School | 10.1 | 7.5 | 7.6 | 6.6 | 4.6 | 0.4 |
|  |  | District | 4.4 | 2.4 | 2.1 | 1.9 | 1.4 | 2.0 |
|  |  | State | 5.2 | 3.9 | 3.5 | 2.8 | 2.5 | 4.2 |
|  | School FRPL: High  (N=13,000) | Non attrition | 73.6 | 80.9 | 82.8 | 84.3 | 86.1 | 92.5 |
|  |  | School | 15.8 | 12.0 | 11.8 | 10.6 | 9.2 | 1.0 |
|  |  | District | 6.3 | 4.0 | 3.1 | 2.9 | 2.5 | 3.4 |
|  |  | State | 4.4 | 3.1 | 2.3 | 2.2 | 2.2 | 3.2 |
| Urbanicity | City  (N=7,000) | Non attrition | 75.5 | 82.8 | 84.8 | 85.9 | 87.8 | 90.7 |
|  |  | School | 16.1 | 10.1 | 9.9 | 9.4 | 7.2 | 0.7 |
|  |  | District | 3.9 | 3.4 | 2.2 | 2.1 | 1.7 | 2.5 |
|  |  | State | 4.5 | 3.8 | 3.1 | 2.7 | 3.3 | 6.1 |
|  | Rural  (N=9,000) | Non attrition | 84.7 | 90.6 | 90.5 | 91.5 | 92.0 | 95.0 |
|  |  | School | 7.6 | 4.3 | 5.3 | 4.6 | 4.2 | 0.2 |
|  |  | District | 3.3 | 1.6 | 1.2 | 1.2 | 1.5 | 1.0 |
|  |  | State | 4.4 | 3.6 | 3.0 | 2.7 | 2.4 | 3.7 |
|  | Suburban  (N=32,000) | Non attrition | 80.6 | 85.3 | 86.8 | 88.6 | 92.0 | 92.8 |
|  |  | School | 9.8 | 8.5 | 7.9 | 6.9 | 4.2 | 0.5 |
|  |  | District | 4.5 | 2.4 | 2.1 | 1.9 | 1.5 | 1.9 |
|  |  | State | 5.1 | 3.8 | 3.3 | 2.7 | 2.4 | 4.8 |
|  | Town  (N=3,000) | Non attrition | 81.1 | 88.1 | 87.9 | 90.1 | 93.7 | >95 |
|  |  | School | 8.5 | 4.6 | 6.4 | 5.0 | 2.8 | <0.1 |
|  |  | District | 5.3 | 2.5 | 2.0 | 1.8 | 1.6 | 2.0 |
|  |  | State | 5.1 | 4.7 | 3.7 | 3.1 | 2.0 | 2.7 |

*Notes.* FRPL = eligibility for free/reduced price meals; ELL = English language learner; SPECED = special education.

Supplemental Table 9. *Annual Attrition for the MS Cohort*

|  |  |  | **Year 1** | **Year 2** | **Year 3** |
| --- | --- | --- | --- | --- | --- |
|  |  |  | (N=52,000) | (N=45,000) | (N=41,000) |
| Total |  | Non attrition | 86.1 | 91.7 | 92.3 |
|  |  | School | 6.9 | 3.9 | 0.7 |
|  |  | District | 3.0 | 1.6 | 1.6 |
|  |  | State | 4.0 | 2.9 | 5.4 |
| Gender | Female  (N=25,000) | Non attrition | 86.6 | 91.8 | 93.0 |
|  |  | School | 6.5 | 3.9 | 0.6 |
|  |  | District | 3.1 | 1.6 | 1.7 |
|  |  | State | 3.9 | 2.8 | 4.7 |
|  | Male  (N=27,000) | Non attrition | 85.6 | 91.5 | 91.7 |
|  |  | School | 7.3 | 3.9 | 0.7 |
|  |  | District | 3.0 | 1.6 | 1.6 |
|  |  | State | 4.1 | 3.0 | 6.0 |
| Race/Ethnicity | Hispanic of Any Race  (N=7,000) | Non attrition | 84.7 | 90.5 | 93.5 |
|  |  | School | 8.7 | 4.7 | 1.0 |
|  |  | District | 2.6 | 1.7 | 1.6 |
|  |  | State | 4.0 | 3.1 | 3.9 |
|  | Non-Hispanic Asian  (N=4,000) | Non attrition | 88.4 | 93.2 | >95 |
|  |  | School | 6.2 | 3.2 | <0.5 |
|  |  | District | 1.4 | 0.7 | 0.9 |
|  |  | State | 4.0 | 3.0 | 3.6 |
|  | Non-Hispanic Black  (N=14,000) | Non attrition | 78.2 | 86.4 | 90.1 |
|  |  | School | 11.8 | 7.7 | 1.4 |
|  |  | District | 6.1 | 3.0 | 3.2 |
|  |  | State | 3.9 | 3.0 | 5.3 |
|  | Non-Hispanic White  (N=25,000) | Non attrition | 90.6 | 94.4 | 92.7 |
|  |  | School | 3.7 | 1.9 | 0.3 |
|  |  | District | 1.8 | 0.9 | 1.0 |
|  |  | State | 4.0 | 2.7 | 6.0 |
|  | Other  (N=3,000) | Non attrition | 85.6 | 90.3 | 91.2 |
|  |  | School | 7.0 | 4.5 | 0.8 |
|  |  | District | 2.8 | 2.2 | 1.7 |
|  |  | State | 4.6 | 3.1 | 6.3 |
| FRPL (Student) | FRPL Students  (N=21,000) | Non attrition | 79.3 | 86.7 | 92.0 |
|  |  | School | 11.4 | 7.4 | 1.4 |
|  |  | District | 5.1 | 2.8 | 2.8 |
|  |  | State | 4.2 | 3.1 | 3.7 |
|  | Non-FRPL Students  (N=31,000) | Non attrition | 90.6 | 94.5 | 92.5 |
|  |  | School | 3.8 | 1.9 | 0.2 |
|  |  | District | 1.7 | 0.9 | 1.0 |
|  |  | State | 3.9 | 2.8 | 6.3 |
| ELL | ELL Students  (N=2,000) | Non attrition | 79.9 | 83.6 | 92.0 |
|  |  | School | 10.7 | 9.0 | 1.7 |
|  |  | District | 2.5 | 1.9 | 1.9 |
|  |  | State | 6.9 | 5.5 | 4.4 |
|  | Non-ELL Students  (N=50,000) | Non attrition | 86.3 | 91.9 | 92.4 |
|  |  | School | 6.7 | 3.7 | 0.6 |
|  |  | District | 3.1 | 1.6 | 1.6 |
|  |  | State | 3.9 | 2.8 | 5.4 |
| SPECED | SPECED students  (N=8,000) | Non attrition | 82.6 | 88.9 | 92.0 |
|  |  | School | 8.9 | 5.5 | 0.9 |
|  |  | District | 4.1 | 2.0 | 2.4 |
|  |  | State | 4.3 | 3.6 | 4.7 |
|  | Non-SPECED students  (N=44,000) | Non attrition | 86.7 | 92.1 | 92.4 |
|  |  | School | 6.5 | 3.6 | 0.6 |
|  |  | District | 2.8 | 1.5 | 1.5 |
|  |  | State | 4.0 | 2.8 | 5.5 |
| FRPL (School) | School FRPL: Low  (N=15,000) | Non attrition | 92.1 | 95.1 | 92.9 |
|  |  | School | 3.1 | 1.7 | 0.2 |
|  |  | District | 1.4 | 0.7 | 0.6 |
|  |  | State | 3.5 | 2.5 | 6.4 |
|  | School FRPL: Medium  (N=27,000) | Non attrition | 85.7 | 91.4 | 92.3 |
|  |  | School | 6.9 | 3.8 | 0.6 |
|  |  | District | 3.1 | 1.7 | 1.8 |
|  |  | State | 4.3 | 3.1 | 5.2 |
|  | School FRPL: High  (N=10,000) | Non attrition | 78.7 | 86.6 | 91.4 |
|  |  | School | 12.0 | 7.9 | 1.5 |
|  |  | District | 5.2 | 2.7 | 3.0 |
|  |  | State | 4.1 | 2.9 | 4.1 |
| Urbanicity | City  (N=9,000) | Non attrition | 83.9 | 89.6 | 92.0 |
|  |  | School | 8.4 | 5.3 | 0.7 |
|  |  | District | 3.0 | 1.5 | 1.6 |
|  |  | State | 4.6 | 3.5 | 5.7 |
|  | Rural  (N=6,000) | Non attrition | 91.1 | 94.3 | 94.4 |
|  |  | School | 2.8 | 2.1 | 0.2 |
|  |  | District | 1.8 | 1.1 | 1.0 |
|  |  | State | 4.2 | 2.4 | 4.5 |
|  | Suburban  (N=34,000) | Non attrition | 85.3 | 91.4 | 91.9 |
|  |  | School | 7.5 | 4.1 | 0.8 |
|  |  | District | 3.3 | 1.7 | 1.8 |
|  |  | State | 3.9 | 2.8 | 5.6 |
|  | Town  (N=2,000) | Non attrition | 92.2 | 95.0 | >94 |
|  |  | School | 1.6 | 1.0 | <0.5 |
|  |  | District | 3.3 | 1.5 | 1.8 |
|  |  | State | 3.0 | 2.5 | 3.8 |

*Notes.* FRPL = eligibility for free/reduced price meals; ELL = English language learner; SPECED = special education.

Supplemental Table 10. *Annual Attrition for the HS Cohort*

|  |  |  | **Year 1** | **Year 2** | **Year 3** | **Year 4** |
| --- | --- | --- | --- | --- | --- | --- |
|  |  |  | (N=70,000) | (N=57,000) | (N=51,000) | (N=45,000) |
| Total |  | Non attrition | 82.1 | 88.5 | 89.5 | 2.5 |
|  |  | School | 7.8 | 4.5 | 2.3 | 0.5 |
|  |  | District | 2.8 | 1.3 | 0.7 | 0.1 |
|  |  | State | 7.4 | 5.3 | 3.9 | 2.3 |
|  |  | High School Completion | 0.0 | 0.3 | 3.7 | 94.5 |
| Gender | Female  (N=33,000) | Non attrition | 83.4 | 89.6 | 90.5 | 1.5 |
|  |  | School | 7.2 | 4.3 | 1.8 | 0.4 |
|  |  | District | 2.7 | 1.3 | 0.7 | 0.0 |
|  |  | State | 6.7 | 4.5 | 3.2 | 1.8 |
|  |  | High School Completion | 0.0 | 0.3 | 3.8 | 96.2 |
|  | Male  (N=37,000) | Non attrition | 80.9 | 87.5 | 88.5 | 3.5 |
|  |  | School | 8.2 | 4.7 | 2.7 | 0.7 |
|  |  | District | 2.8 | 1.4 | 0.7 | 0.1 |
|  |  | State | 8.1 | 6.0 | 4.5 | 2.9 |
|  |  | High School Completion | 0.0 | 0.3 | 3.6 | 92.9 |
| Race/Ethnicity | Hispanic of Any Race  (N=11,000) | Non attrition | 79.5 | 83.8 | 82.6 | 5.5 |
|  |  | School | 7.4 | 4.8 | 2.4 | 1.0 |
|  |  | District | 2.3 | 1.5 | 0.7 | 0.1 |
|  |  | State | 10.8 | 9.4 | 8.0 | 5.1 |
|  |  | High School Completion | 0.0 | 0.5 | 6.3 | 88.3 |
|  | Non-Hispanic Asian  (N=4,000) | Non attrition | 89.5 | 94.2 | 94.9 | 1.4 |
|  |  | School | 4.5 | 1.7 | 0.7 | <0.5 |
|  |  | District | 1.6 | 0.6 | 0.5 | 0.0 |
|  |  | State | 4.5 | 3.1 | 1.4 | <1 |
|  |  | High School Completion | 0.0 | 0.5 | 2.6 | 98.0 |
|  | Non-Hispanic Black  (N=25,000) | Non attrition | 73.3 | 84.7 | 85.8 | 3.6 |
|  |  | School | 13.2 | 7.1 | 3.9 | 0.9 |
|  |  | District | 4.5 | 2.2 | 1.0 | 0.1 |
|  |  | State | 9.0 | 5.5 | 3.9 | 2.6 |
|  |  | High School Completion | 0.0 | 0.5 | 5.3 | 92.8 |
|  | Non-Hispanic White  (N=27,000) | Non attrition | 89.8 | 92.1 | 93.3 | 1.3 |
|  |  | School | 3.5 | 3.0 | 1.5 | 0.3 |
|  |  | District | 1.5 | 0.7 | 0.4 | 0.0 |
|  |  | State | 5.3 | 4.0 | 2.9 | 1.7 |
|  |  | High School Completion | 0.0 | 0.1 | 1.9 | 96.7 |
|  | Other  (N=3,000) | Non attrition | 84.9 | >89 | 90.9 | 1.1 |
|  |  | School | 7.3 | 4.2 | 1.5 | <0.5 |
|  |  | District | 2.8 | 1.1 | 0.9 | <0.5 |
|  |  | State | 5.1 | 5.3 | 3.2 | 2.3 |
|  |  | High School Completion | 0.0 | <0.5 | 3.5 | >96 |
| FRPL (Student) | FRPL Students  (N=30,000) | Non attrition | 74.4 | 82.1 | 82.9 | 4.8 |
|  |  | School | 12.0 | 7.5 | 3.8 | 1.1 |
|  |  | District | 4.1 | 2.3 | 1.2 | 0.1 |
|  |  | State | 9.5 | 7.6 | 6.3 | 4.7 |
|  |  | High School Completion | 0.0 | 0.5 | 5.9 | 89.3 |
|  | Non-FRPL Students  (N=39,000) | Non attrition | 87.9 | 92.6 | 93.3 | 1.4 |
|  |  | School | 4.5 | 2.6 | 1.4 | 0.3 |
|  |  | District | 1.8 | 0.7 | 0.4 | 0.0 |
|  |  | State | 5.8 | 3.8 | 2.5 | 1.2 |
|  |  | High School Completion | 0.0 | 0.2 | 2.5 | 97.2 |
| ELL | ELL Students  (N=5,000) | Non attrition | 73.1 | 74.8 | 71.8 | 11.4 |
|  |  | School | 8.9 | 6.2 | 2.3 | 1.5 |
|  |  | District | 2.4 | 1.7 | 0.9 | 0.2 |
|  |  | State | 15.6 | 16.1 | 15.2 | 10.7 |
|  |  | High School Completion | 0.0 | 1.2 | 9.7 | 76.2 |
|  | Non-ELL Students  (N=65,000) | Non attrition | 82.7 | 89.4 | 90.4 | 2.2 |
|  |  | School | 7.7 | 4.4 | 2.3 | 0.5 |
|  |  | District | 2.8 | 1.3 | 0.7 | 0.1 |
|  |  | State | 6.8 | 4.6 | 3.3 | 2.0 |
|  |  | High School Completion | 0.0 | 0.3 | 3.4 | 95.3 |
| SPECED | SPECED students  (N=11,000) | Non attrition | 75.4 | 84.6 | 85.9 | 9.9 |
|  |  | School | 11.9 | 6.4 | 3.3 | 1.4 |
|  |  | District | 3.7 | 1.7 | 1.0 | 0.2 |
|  |  | State | 9.1 | 7.0 | 5.4 | 4.5 |
|  |  | High School Completion | 0.0 | 0.4 | 4.4 | 84.1 |
|  | Non-SPECED students  (N=59,000) | Non attrition | 83.3 | 89.2 | 90.1 | 1.4 |
|  |  | School | 7.0 | 4.2 | 2.1 | 0.4 |
|  |  | District | 2.6 | 1.3 | 0.6 | 0.0 |
|  |  | State | 7.1 | 5.0 | 3.6 | 2.0 |
|  |  | High School Completion | 0.0 | 0.3 | 3.6 | 96.1 |
| FRPL (School) | School FRPL: Low  (N=17,000) | Non attrition | 89.6 | 94.5 | 93.9 | 1.1 |
|  |  | School | 4.3 | 1.6 | 1.6 | 0.2 |
|  |  | District | 1.4 | 0.5 | 0.3 | 0.0 |
|  |  | State | 4.7 | 3.3 | 2.2 | 1.0 |
|  |  | High School Completion | 0.0 | 0.1 | 2.0 | 97.7 |
|  | School FRPL: Medium  (N=38,000) | Non attrition | 82.9 | 88.0 | 89.6 | 2.5 |
|  |  | School | 7.4 | 4.8 | 1.9 | 0.4 |
|  |  | District | 2.7 | 1.5 | 0.7 | 0.1 |
|  |  | State | 7.0 | 5.3 | 3.9 | 2.3 |
|  |  | High School Completion | 0.0 | 0.4 | 4.0 | 94.8 |
|  | School FRPL: High  (N=14,000) | Non attrition | 71.3 | 81.2 | 81.8 | 5.7 |
|  |  | School | 12.6 | 7.9 | 4.8 | 1.6 |
|  |  | District | 4.6 | 2.2 | 1.3 | 0.1 |
|  |  | State | 11.5 | 8.2 | 6.5 | 5.1 |
|  |  | High School Completion | 0.0 | 0.5 | 5.7 | 87.6 |
| Urbanicity | City  (N=15,000) | Non attrition | 78.1 | 85.6 | 89.7 | 3.9 |
|  |  | School | 10.2 | 6.7 | 2.2 | 0.8 |
|  |  | District | 2.9 | 1.5 | 0.9 | 0.0 |
|  |  | State | 8.8 | 6.0 | 4.3 | 3.2 |
|  |  | High School Completion | 0.0 | 0.3 | 2.9 | 92.1 |
|  | Rural  (N=11,000) | Non attrition | 87.9 | 92.9 | 93.9 | 1.4 |
|  |  | School | 5.0 | 2.3 | 0.7 | 0.2 |
|  |  | District | 2.1 | 1.0 | 0.5 | 0.0 |
|  |  | State | 5.0 | 3.8 | 2.5 | 1.6 |
|  |  | High School Completion | 0.0 | 0.1 | 2.4 | 96.8 |
|  | Suburban  (N=42,000) | Non attrition | 81.5 | 87.9 | 87.8 | 2.5 |
|  |  | School | 7.9 | 4.6 | 2.9 | 0.6 |
|  |  | District | 3.0 | 1.4 | 0.7 | 0.1 |
|  |  | State | 7.7 | 5.6 | 4.1 | 2.3 |
|  |  | High School Completion | 0.0 | 0.4 | 4.5 | 94.5 |
|  | Town  (N=2,000) | Non attrition | 92.3 | 94.5 | 93.9 | 1.2 |
|  |  | School | 1.9 | 1.0 | 0.7 | <0.6 |
|  |  | District | 2.1 | 1.0 | 0.6 | 0.0 |
|  |  | State | 3.8 | 3.6 | 3.3 | 1.9 |
|  |  | High School Completion | 0.0 | 0.1 | 1.5 | >96 |

*Notes.* FRPL = eligibility for free/reduced price meals; ELL = English language learner; SPECED = special education.

Supplemental Table 11. *Annual Attrition for the PS-HSG Associate Degree Cohort*

|  | |  | **Year 1** | **Year 2** | **Year 3** |
| --- | --- | --- | --- | --- | --- |
|  |  |  | (N=13,000) | (N=9,000) | (N=5,000) |
| Total |  | Obtained degree | 0.2 | 7.1 | 20.0 |
|  |  | No Attrition | 66.4 | 61.8 | 45.7 |
|  |  | College | 2.9 | 1.8 | 1.9 |
|  |  | System | 5.9 | 8.3 | 11.4 |
|  |  | State | 3.1 | 3.0 | 2.0 |
|  |  | Postsecondary | 21.6 | 18.0 | 18.9 |
| Gender | Female  (N=7,000) | Obtained degree | 0.2 | 7.6 | 20.7 |
|  |  | No Attrition | 67.6 | 61.5 | 46.0 |
|  |  | College | 3.0 | 2.2 | 2.2 |
|  |  | System | 6.0 | 7.7 | 10.2 |
|  |  | State | 3.2 | 3.3 | 2.2 |
|  |  | Postsecondary | 20.0 | 17.7 | 18.6 |
|  | Male  (N=6,000) | Obtained degree | 0.2 | 6.6 | 19.3 |
|  |  | No Attrition | 65.0 | 62.2 | 45.4 |
|  |  | College | 2.9 | 1.2 | 1.6 |
|  |  | System | 5.7 | 9.0 | 12.6 |
|  |  | State | 3.0 | 2.6 | 1.8 |
|  |  | Postsecondary | 23.3 | 18.4 | 19.3 |
| Race/Ethnicity | Hispanic of Any Race  (N=1,000) | Obtained degree | <1 | 4.7 | 16.3 |
|  |  | No Attrition | 76.5 | 70.4 | 52.7 |
|  |  | College | 2.7 | 1.4 | <2 |
|  |  | System | 2.9 | 6.6 | 11.1 |
|  |  | State | <3 | 2.1 | <2 |
|  |  | Postsecondary | 15.6 | 14.7 | 18.6 |
|  | Non-Hispanic Asian  (N=1,000) | Obtained degree | 0.0 | 8.2 | 18.6 |
|  |  | No Attrition | 80.4 | 67.8 | 47.1 |
|  |  | College | 1.4 | <2 | <3 |
|  |  | System | 8.9 | 11.8 | 19.3 |
|  |  | State | 1.8 | <2 | <3 |
|  |  | Postsecondary | 7.6 | 10.1 | 12.3 |
|  | Non-Hispanic Black  (N=4,000) | Obtained degree | <1 | 1.8 | 10.1 |
|  |  | No Attrition | 57.6 | 59.8 | 46.7 |
|  |  | College | 4.2 | 2.8 | 3.1 |
|  |  | System | 5.2 | 7.0 | 12.0 |
|  |  | State | <3 | 2.9 | 2.1 |
|  |  | Postsecondary | 30.1 | 25.7 | 26.0 |
|  | Non-Hispanic White  (N=6,000) | Obtained degree | 0.2 | 10.5 | 26.7 |
|  |  | No Attrition | 68.0 | 59.5 | 43.2 |
|  |  | College | 2.3 | 1.4 | 1.7 |
|  |  | System | 6.4 | 9.0 | 9.4 |
|  |  | State | 3.6 | 3.5 | 2.5 |
|  |  | Postsecondary | 19.5 | 16.2 | 16.6 |
|  | Other  (N=1,000) | Obtained degree | <1 | 7.5 | 22.6 |
|  |  | No Attrition | 67.5 | 65.9 | 43.8 |
|  |  | College | 3.4 | <3 | <3 |
|  |  | System | 7.6 | 7.8 | 14.5 |
|  |  | State | <4 | <3 | <3 |
|  |  | Postsecondary | 18.0 | 14.2 | 15.5 |

Supplemental Table 12. *Annual Attrition for the PS-HSG Bachelor’s Degree Cohort*

|  | |  | **Year 1** | **Year 2** | **Year 3** | **Year 4** | **Year 5** | **Year 6** |
| --- | --- | --- | --- | --- | --- | --- | --- | --- |
|  |  |  | (N=10,000) | (N=8,000) | (N=7,000) | (N=6,000) | (N=2,000) | (N<500) |
| Total |  | Obtained degree | 0.0 | 0.0 | 1.1 | 60.4 | 67.0 | 54.2 |
|  |  | No Attrition | 75.0 | 86.6 | 88.3 | 31.0 | 22.3 | 28.0 |
|  |  | College | 2.1 | 1.8 | 1.3 | 0.6 | <1 | <3 |
|  |  | System | 17.9 | 6.7 | 5.7 | 2.3 | 1.2 | <3 |
|  |  | State | 1.5 | 1.0 | 0.9 | 0.4 | <1 | <3 |
|  |  | Postsecondary | 3.6 | 3.7 | 2.8 | 5.4 | 8.7 | 16.1 |
| Gender | Female  (n=6,000) | Obtained degree | 0.0 | 0.0 | 1.2 | 65.7 | 70.2 | 56.7 |
|  |  | No Attrition | 74.4 | 86.3 | 87.7 | 27.1 | 20.6 | 28.1 |
|  |  | College | 2.0 | 2.0 | 1.3 | 0.6 | <1 | <6 |
|  |  | System | 19.0 | 7.2 | 6.6 | 2.1 | 1.3 | <6 |
|  |  | State | 1.6 | 1.1 | 1.1 | 0.4 | <1 | <6 |
|  |  | Postsecondary | 3.0 | 3.4 | 2.2 | 4.2 | 7.4 | 12.9 |
|  | Male  (n=5,000) | Obtained degree | 0.0 | 0.0 | 0.9 | 54.0 | 64.1 | 52.2 |
|  |  | No Attrition | 75.8 | 87.1 | 89.0 | 35.7 | 23.8 | 27.9 |
|  |  | College | 2.1 | 1.6 | 1.3 | 0.6 | <1 | <5 |
|  |  | System | 16.5 | 6.1 | 4.6 | 2.5 | 1.2 | <5 |
|  |  | State | 1.3 | 1.0 | 0.6 | 0.4 | <1 | 0.0 |
|  |  | Postsecondary | 4.3 | 4.1 | 3.6 | 6.8 | 9.8 | 18.6 |
| Race/Ethnicity | Hispanic of Any Race  (n=1000) | Obtained degree | 0.0 | 0.0 | <3 | 54.2 | 69.9 | 68.0 |
|  |  | No Attrition | 73.5 | 90.0 | 88.1 | 35.9 | 18.8 | <20 |
|  |  | College | 1.1 | <3 | <3 | <3 | 0.0 | 0.0 |
|  |  | System | 20.4 | 4.3 | 5.9 | <3 | <10 | 0.0 |
|  |  | State | 1.6 | <3 | <3 | <3 | 0.0 | 0.0 |
|  |  | Postsecondary | 3.5 | 2.8 | 3.3 | 6.7 | <10 | <20 |
|  | Non-Hispanic Asian  (n=1000) | Obtained degree | <1 | 0.0 | <2 | 67.3 | 70.5 | 60.0 |
|  |  | No Attrition | 76.0 | 90.5 | 90.3 | 26.2 | 19.3 | <28 |
|  |  | College | 2.9 | <2 | <2 | <2 | <5 | <28 |
|  |  | System | 19.2 | 4.1 | 4.6 | 2.0 | <5 | 0.0 |
|  |  | State | <1 | <2 | <2 | <2 | 0.0 | 0.0 |
|  |  | Postsecondary | <1 | 2.8 | <2 | 3.8 | 8.7 | <28 |
|  | Non-Hispanic Black  (n=3000) | Obtained degree | 0.0 | 0.0 | <1 | 40.5 | 59.6 | 50.5 |
|  |  | No Attrition | 74.1 | 80.5 | 85.5 | 46.5 | 28.2 | 30.1 |
|  |  | College | 2.3 | 2.7 | <2 | 0.9 | <1 | <2 |
|  |  | System | 15.6 | 8.9 | 6.8 | 4.3 | 1.5 | <2 |
|  |  | State | 1.5 | 1.4 | <2 | 0.8 | <1 | 0.0 |
|  |  | Postsecondary | 6.5 | 6.6 | 5.4 | 7.0 | 9.5 | 17.5 |
|  | Non-Hispanic White  (n=5000) | Obtained degree | 0.0 | 0.0 | 1.5 | 70.0 | 73.8 | 54.9 |
|  |  | No Attrition | 75.5 | 89.0 | 89.5 | 23.5 | 16.9 | 28.3 |
|  |  | College | 1.9 | 1.2 | 1.4 | <1 | <1 | 0.0 |
|  |  | System | 18.3 | 6.4 | 5.2 | 1.2 | <1 | 0.0 |
|  |  | State | 1.6 | 0.9 | 0.7 | <1 | <1 | <10 |
|  |  | Postsecondary | 2.6 | 2.5 | 1.6 | 4.6 | 7.6 | <20 |
|  | Other  (n=500) | Obtained degree | 0.0 | 0.0 | <4 | 62.9 | 64.0 | 60.0 |
|  |  | No Attrition | 75.3 | 88.0 | 87.0 | 26.8 | 26.7 | 30.0 |
|  |  | College | <2 | <3 | <4 | <4 | 0.0 | 0.0 |
|  |  | System | 21.0 | 6.8 | 6.8 | <4 | <10 | <10 |
|  |  | State | <2 | <3 | <4 | 0.0 | 0.0 | 0.0 |
|  |  | Postsecondary | <2 | <3 | <4 | 6.8 | <10 | <10 |
| Postsecondary System | 4-Year State-Aided (MICUA; n=1500) | Obtained degree | 0.0 | 0.0 | <2 | 81.2 | 72.5 | * |
|  |  | No Attrition | 71.7 | 83.6 | 85.2 | 12.2 | <13 | * |
|  |  | College | <1 | <1 | <1 | 0.0 | 0.0 | 0.0 |
|  |  | System | 22.1 | 11.5 | 10.0 | <3 | <13 | * |
|  |  | State | <3 | <2 | <2 | <2 | 0.0 | 0.0 |
|  |  | Postsecondary | 3.3 | 3.2 | <2 | 4.1 | 13.2 | * |
|  | Morgan  (n=500) | Obtained degree | 0.0 | 0.0 | 0.0 | 24.2 | 54.4 | 39.6 |
|  |  | No Attrition | 78.2 | 79.6 | 84.0 | 63.5 | 33.1 | 41.5 |
|  |  | College | N/A | N/A | N/A | N/A | N/A | N/A |
|  |  | System | 11.8 | 11.1 | 7.7 | <5 | <7 | <20 |
|  |  | State | <3 | <3 | <4 | <5 | 0.0 | 0.0 |
|  |  | Postsecondary | <10 | <10 | <7 | 7.1 | <12 | <20 |
|  | St. Mary’s (n=300) | Obtained degree | 0.0 | 0.0 | <6 | 86.6 | 85.7 | * |
|  |  | No Attrition | 77.5 | 87.8 | 86.6 | 9.4 | <15 | 0.0 |
|  |  | College | N/A | N/A | N/A | N/A | N/A | N/A |
|  |  | System | 19.8 | 9.2 | 10.5 | <7 | <15 | 0.0 |
|  |  | State | <3 | <6 | <6 | 0.0 | 0.0 | 0.0 |
|  |  | Postsecondary | <3 | <6 | <6 | <7 | 0.0 | 0.0 |
|  | 4-Year Public (USM; n=8000) | Obtained degree | 0.0 | 0.0 | 1.1 | 58.2 | 67.9 | 57.2 |
|  |  | No Attrition | 75.4 | 87.6 | 89.1 | 32.9 | 21.9 | 26.3 |
|  |  | College | 2.5 | 2.3 | 1.5 | 0.7 | <1 | <3 |
|  |  | System | 17.4 | 5.5 | 4.7 | 2.1 | 1.1 | <3 |
|  |  | State | 1.3 | 0.9 | 0.7 | 0.5 | <1 | <3 |
|  |  | Postsecondary | 3.4 | 3.7 | 2.9 | 5.6 | 8.2 | 15.3 |

Supplemental Table 13. *Annual Attrition for the PS-MD Associate Degree Cohort*

|  | |  | **Year 1** | **Year 2** | **Year 3** |
| --- | --- | --- | --- | --- | --- |
|  |  |  | (N=23,000) | (N=14,000) | (N=8,000) |
| Total |  | Obtained degree | 0.3 | 5.9 | 18.0 |
|  |  | No Attrition | 59.0 | 61.5 | 47.4 |
|  |  | College | 2.9 | 1.7 | 2.0 |
|  |  | System | 5.1 | 6.8 | 10.4 |
|  |  | State | 32.7 | 24.2 | 22.3 |
| Gender | Female  (n=12,000) | Obtained degree | 0.3 | 6.00 | 18.1 |
|  |  | No Attrition | 60.8 | 62.2 | 48.8 |
|  |  | College | 3.0 | 1.9 | 2.1 |
|  |  | System | 5.2 | 6.2 | 9.5 |
|  |  | State | 30.7 | 23.72 | 21.6 |
|  | Male  (n=11,000) | Obtained degree | 0.3 | 5.7 | 17.9 |
|  |  | No Attrition | 57.2 | 60.7 | 45.6 |
|  |  | College | 2.8 | 1.4 | 1.8 |
|  |  | System | 5.1 | 7.6 | 11.6 |
|  |  | State | 34.6 | 24.7 | 23.1 |
| Race/Ethnicity | Hispanic of Any Race  (n=2,000) | Obtained degree | <0.6 | 4.3 | 15.8 |
|  |  | No Attrition | 67.2 | 68.0 | 50.8 |
|  |  | College | <3 | 1.3 | 1.4 |
|  |  | System | 3.2 | 5.6 | 10.2 |
|  |  | State | 26.8 | 20.8 | 21.9 |
|  | Non-Hispanic Asian  (n=1,000) | Obtained degree | <0.2 | <7 | 18.8 |
|  |  | No Attrition | 73.7 | 67.8 | 47.1 |
|  |  | College | <2 | <0.2 | 2.1 |
|  |  | System | 7.2 | 10.3 | 16.7 |
|  |  | State | 17.1 | 14.5 | 15.4 |
|  | Non-Hispanic Black  (n=8,000) | Obtained degree | 0.2 | 1.8 | 9.2 |
|  |  | No Attrition | 51.6 | 59.7 | 50.5 |
|  |  | College | 3.8 | 2.4 | 2.6 |
|  |  | System | 4.4 | 5.5 | 11.2 |
|  |  | State | 40.1 | 30.7 | 26.5 |
|  | Non-Hispanic White  (11,000) | Obtained degree | 0.4 | 8.8 | 23.8 |
|  |  | No Attrition | 61.4 | 59.9 | 44.9 |
|  |  | College | 2.3 | 1.4 | 1.6 |
|  |  | System | 5.8 | 7.4 | 8.8 |
|  |  | State | 30.1 | 22.5 | 20.9 |
|  | Other  (n=1,000) | Obtained degree | <2 | 6.2 | 21.2 |
|  |  | No Attrition | 62.7 | 65.5 | 42.9 |
|  |  | College | <4 | 1.9 | 2.7 |
|  |  | System | 6.1 | 7.4 | 12.6 |
|  |  | State | 27.9 | 19.0 | 20.6 |

*Notes. N* = 38 students are missing gender information; *N* = 60 students are missing race/ethnicity information.

Supplemental Table 14. *Annual Attrition for the PS-MD Bachelor’s Degree Cohort (N =20,000)*

|  | |  | **Year 1** | **Year 2** | **Year 3** | **Year 4** | **Year 5** | **Year 6** |
| --- | --- | --- | --- | --- | --- | --- | --- | --- |
|  |  |  | (N=20,000) | (N=16,000) | (N=14,000) | (N=12,000) | (N=3,000) | (N=600) |
| Total |  | Obtained degree | 0.0 | 0.0 | 1.4 | 69.1 | 67.9 | 51.2 |
|  |  | No Attrition | 77.1 | 87.9 | 90.2 | 23.6 | 20.3 | 28.0 |
|  |  | College | 1.5 | 1.3 | 1.0 | 0.4 | 0.6 | <2 |
|  |  | System | 11.8 | 4.7 | 4.0 | 1.4 | 1.2 | <2 |
|  |  | State | 9.7 | 6.1 | 3.4 | 5.5 | 10.0 | 19.1 |
| Gender | Female  (n=11,000) | Obtained degree | 0.0 | 0.0 | 1.6 | 73.7 | 70.0 | >50 |
|  |  | No Attrition | 76.8 | 87.9 | 90.3 | 20.1 | 18.7 | 28.4 |
|  |  | College | 1.4 | 1.5 | 1.0 | 0.4 | <1 | <5 |
|  |  | System | 12.3 | 5.0 | 4.5 | 1.3 | <2 | <5 |
|  |  | State | 9.5 | 5.6 | 2.7 | 4.5 | 9.5 | 17.3 |
|  | Male  (n=9,000) | Obtained degree | 0.0 | 0.0 | 1.1 | 63.6 | 66.1 | >50 |
|  |  | No Attrition | 77.6 | 87.9 | 90.2 | 27.8 | 21.7 | 27.8 |
|  |  | College | 1.6 | 1.1 | 1.0 | 0.4 | <1 | <4 |
|  |  | System | 11.1 | 4.3 | 3.5 | 1.6 | 1.1 | <4 |
|  |  | State | 9.8 | 6.6 | 4.2 | 6.7 | 10.5 | 20.4 |
| Race/Ethnicity | Hispanic of Any Race  (n=1,000) | Obtained degree | 0.0 | 0.0 | <2 | 68.2 | 68.2 | 55.8 |
|  |  | No Attrition | 77.5 | 89.1 | 90.7 | 23.9 | 19.6 | <25 |
|  |  | College | <1 | 1.1 | <1 | <1 | <5 | 0.0 |
|  |  | System | 12.1 | 3.3 | 3.6 | 1.5 | <5 | 0.0 |
|  |  | State | <10 | 6.5 | 3.5 | 6.0 | 10.9 | <25 |
|  | Non-Hispanic Asian  (n=2,000) | Obtained degree | <1 | 0.0 | 2.4 | 72.2 | 73.1 | 53.7 |
|  |  | No Attrition | 81.0 | 91.9 | 90.6 | 22.3 | 17.5 | 25.9 |
|  |  | College | <2.0 | 1.3 | 1.2 | <1 | <4 | <20 |
|  |  | System | 13.5 | 3.6 | 3.8 | <2 | <4 | 0.0 |
|  |  | State | 3.4 | 3.3 | 2.0 | 3.8 | 8.4 | <20 |
|  | Non-Hispanic Black  (n=5,000) | Obtained degree | 0.0 | 0.0 | 0.9 | 45.7 | 59.2 | 48.5 |
|  |  | No Attrition | 70.7 | 80.7 | 86.1 | 42.7 | 27.2 | 30.4 |
|  |  | College | 1.9 | 2.3 | 1.2 | 0.7 | <1 | <4 |
|  |  | System | 12.5 | 7.3 | 5.4 | 3.3 | <2 | <4 |
|  |  | State | 14.9 | 9.7 | 6.4 | 7.6 | 11.0 | 18.8 |
|  | Non-Hispanic White  (n=10,000) | Obtained degree | 0.0 | 0.0 | 1.4 | 77.3 | 74.8 | 52.9 |
|  |  | No Attrition | 79.6 | 90.4 | 91.7 | 17.0 | 15.1 | 27.3 |
|  |  | College | 1.3 | 0.9 | 1.0 | 0.3 | <1 | <6 |
|  |  | System | 11.2 | 4.0 | 3.6 | 0.7 | <1 | 0.0 |
|  |  | State | 7.9 | 4.7 | 2.3 | 4.7 | 8.9 | <20 |
|  | Other  (n=1,000) | Obtained degree | <1 | 0.0 | <2 | 69.4 | 67.3 | 56.7 |
|  |  | No Attrition | 80.9 | 88.2 | 91.0 | 20.7 | 18.2 | <37 |
|  |  | College | <1 | 1.3 | <2 | <2 | <7 | 0.0 |
|  |  | System | 11.2 | 4.6 | 3.7 | <2 | <7 | <37 |
|  |  | State | 7.1 | 5.8 | 3.8 | 8.2 | 13.3 | <37 |
| Postsecondary System | 4-Year State-Aided (MICUA; n=6,000) | Obtained degree | 0.0 | 0.0 | 2.0 | 86.6 | 72.0 | 46.0 |
|  |  | No Attrition | 82.0 | 89.8 | 91.5 | 8.1 | 11.3 | <30 |
|  |  | College | 0.3 | 0.2 | 0.3 | 0.0 | <4 | 0.0 |
|  |  | System | 8.6 | 4.5 | 3.8 | 0.7 | <4 | <30 |
|  |  | State | 9.1 | 5.4 | 2.5 | 4.6 | 14.9 | <30 |
|  | Morgan  (n=900) | Obtained degree | 0.0 | 0.0 | <2 | 29.9 | 56.8 | 41.0 |
|  |  | No Attrition | 75.2 | 80.3 | 85.6 | 59.6 | 30.4 | 39.8 |
|  |  | College | N/A | N/A | N/A | N/A | N/A | N/A |
|  |  | System | 8.2 | 7.5 | <6 | 3.7 | <4 | <14 |
|  |  | State | 16.6 | 12.2 | 8.6 | 6.8 | <12 | <17 |
|  | St. Mary’s  (n=400) | Obtained degree | 0.0 | 0.0 | <5 | 86.2 | 72.7 | * |
|  |  | No Attrition | 76.7 | 89.7 | 88.9 | 9.5 | <50 | * |
|  |  | College | N/A | N/A | N/A | N/A | N/A | N/A |
|  |  | System | 19.6 | <8 | 8.5 | <5 | <50 | 0.0 |
|  |  | State | 3.7 | <4 | <5 | <5 | <50 | * |
|  | 4-Year Public (USM; n=13,000) | Obtained degree | 0.0 | 0.0 | 1.2 | 61.5 | 68.6 | 53.5 |
|  |  | No Attrition | 75.0 | 87.4 | 89.9 | 30.2 | 20.5 | 26.2 |
|  |  | College | 2.2 | 2.0 | 1.5 | 0.6 | 0.7 | <3 |
|  |  | System | 13.2 | 4.6 | 3.9 | 1.6 | 1.1 | <3 |
|  |  | State | 9.6 | 6.1 | 3.5 | 6.0 | 9.1 | 18.8 |

*Notes. N* = 11 students are missing gender information; *N* = 108 students are missing race/ethnicity information.

Supplemental Table 15. *Descriptive Statistics for the ES, MS, and HS Cohorts*

|  | Elementary School Cohort  (N = 51,000) | Middle School Cohort  (N = 52,000) | High School Cohort  (N = 70,000) |
| --- | --- | --- | --- |
| Student Level Covariates | N (%) | N (%) | N (%) |
| Gender (Female) | 25,000 (48) | 25,000 (49) | 33,000 (47) |
| Race/Ethnicity |  |  |  |
| Hispanic of Any Race | 7,000 (14) | 7,000 (13) | 11,000 (16) |
| Non-Hispanic Asian | 4,000 (7) | 4,000 (7) | 4,000 (6) |
| Non-Hispanic Black | 14,000 (27) | 14,000 (27) | 25,000 (35) |
| Non-Hispanic White | 24,000 (46) | 25,000 (48) | 27,000 (39) |
| Other | 3,000 (6) | 3,000 (5) | 3,000 (4) |
| FRPL (Yes) | 23,000 (44) | 21,000 (40) | 30,000 (43) |
| ELL (Yes) | 7,000 (13) | 2,000 (4) | 5,000 (7) |
| Special Ed (Yes) | 5,000 (10) | 8,000 (16) | 11,000 (16) |
| School Level Covariates | N (%) | N (%) | N (%) |
| FRPL |  |  |  |
| School FRPL: Low | 13,000 (26) | 15,000 (28) | 17,000 (24) |
| School FRPL: Medium | 25,000 (49) | 27,000 (52) | 38,000 (55) |
| School FRPL: High | 13,000 (25) | 10,000 (20) | 14,000 (21) |
| Urbanicity |  |  |  |
| City | 7,000 (14) | 9,000 (17) | 15,000 (21) |
| Rural | 9,000 (18) | 6,000 (12) | 11,000 (15) |
| Suburban | 32,000 (63) | 34,000 (67) | 42,000 (60) |
| Town | 3,000 (5) | 2,000 (4) | 2,000 (3) |

*Notes.* FRPL = eligibility for free/reduced price meals. ELL = English Language Learner*.*

Supplemental Table 16. *Descriptive Statistics for the PS-MD and PS-HSG Cohorts*

|  | *PS-MD* – Assoc (N=23,000) | *PS-MD* – Bach (N=20,000) | *PS-HSG* – Assoc (N=13,000) | *PS-HSG* – Bach (N=10,000) |
| --- | --- | --- | --- | --- |
| Student Level Covariates | N (%) | N (%) | N (%) | N (%) |
| Gender (Female) | 12,000 (53) | 11,000 (55) | 7,000 (52) | 6,000 (56) |
| Race/Ethnicity |  |  |  |  |
| Hispanic of Any Race | 2,000 (10) | 1,000 (7) | 1,000 (10) | 1,000 (6) |
| Non-Hispanic Asian | 1,000 (5) | 2,000 (10) | 1,000 (6) | 1,000 (12) |
| Non-Hispanic Black | 8,000 (35) | 5,000 (26) | 4,000 (31) | 3,000 (30) |
| Non-Hispanic White | 11,000 (45) | 10,000 (51) | 6,000 (47) | 5,000 (46) |
| Other | 1,000 (5) | 1,000 (6) | 1,000 (5) | 500 (5) |
| College Level Covariates |  |  |  |  |
| Postsecondary System |  |  |  |  |
| Community College | 23,000 (100) | N/A | 13,000 (100) | N/A |
| 4-Year State-Aided (MICUA) | <11 (<1) | 6,000 (30) | <11 (<1) | 1,500 (14) |
| Morgan | N/A | 900 (4) | N/A | 500 (5) |
| St. Mary’s | N/A | 400 (2) | N/A | 300 (2) |
| 4-Year Public (USM) | 31 (<1) | 13,000 (64) | 0 | 8,000 (78) |

*Notes.* For the PS-MD Cohort – Associate 38 individuals were missing gender and 60 individuals were missing race/ethnicity; for the PS-MD Cohort – Bachelor’s 11 were missing gender and 108 were missing race/ethnicity.

Supplemental Table 17. *Descriptive Statistics for the Subset of the Sample with Fifth Grade Test Scores (Sample Used for Outcome Analyses)*

|  | *N* (%) |
| --- | --- |
| Gender (Female) | 21,400 (49) |
| Race/Ethnicity |  |
| Hispanic of Any Race | 5,577 (12) |
| Non-Hispanic Asian | 3,189 (7) |
| Non-Hispanic Black | 11,641 (27) |
| Non-Hispanic White | 20,842 (48) |
| Other | 2,334 (5) |
| FRPL (Yes) | 17,479 (40) |
| ELL (Yes) | 1,242 (3) |
| Special Ed (Yes) | 6,512 (15) |
| *Notes*. FRPL = eligibility for free/reduced price meals. ELL = English Language Learner. Total *N* = 43,583. | |

Supplemental Fig. 1. *Cumulative Attrition for PS-HSG Associate Degree Cohort*

*Notes.* For the PS-HSG and PS-MD cohorts: no attrition = student remained in the same college; attrite from college = student moved to a different college within the same postsecondary system; attrite from system = student moved to a different college in a different postsecondary system; attrite from state = student left the Maryland higher education system. For the PS-HSG cohorts: postsecondary = student left the postsecondary system all together (i.e., was not found in a Maryland institution or an out-of-state institution—determined using data from the National Student Clearinghouse).

Supplemental Fig. 2. *Cumulative Attrition for PS-HSG Bachelor’s Degree Cohort*

*Notes.* For the PS-HSG and PS-MD cohorts: no attrition = student remained in the same college; attrite from college = student moved to a different college within the same postsecondary system; attrite from system = student moved to a different college in a different postsecondary system; attrite from state = student left the Maryland higher education system. For the PS-HSG cohorts: postsecondary = student left the postsecondary system all together (i.e., was not found in a Maryland institution or an out-of-state institution—determined using data from the National Student Clearinghouse).

Supplemental Fig. 3. *Cumulative Attrition for the PS-MD Associate Degree Cohort*

*Notes.* For the PS-HSG and PS-MD cohorts: no attrition = student remained in the same college; attrite from college = student moved to a different college within the same postsecondary system; attrite from system = student moved to a different college in a different postsecondary system; attrite from state = student left the Maryland higher education system.

Supplemental Fig. 4. *Cumulative Attrition for the PS-MD Bachelor’s Degree Cohort*

*Notes.* For the PS-HSG and PS-MD cohorts: no attrition = student remained in the same college; attrite from college = student moved to a different college within the same postsecondary system; attrite from system = student moved to a different college in a different postsecondary system; attrite from state = student left the Maryland higher education system.
